# Associations of interactions between *NLRP3* SNPs and HLA mismatch with acute and extensive chronic graft-versus-host diseases

**DOI:** 10.1038/s41598-017-13506-w

**Published:** 2017-10-12

**Authors:** Hidekazu Takahashi, Naoko Okayama, Natsu Yamaguchi, Yuta Miyahara, Yasuo Morishima, Yutaka Suehiro, Takahiro Yamasaki, Koji Tamada, Satoshi Takahashi, Arinobu Tojo, Shigetaka Asano, Tsuyoshi Tanabe

**Affiliations:** 1 Department of Public Health and Preventive Medicine, Yamaguchi University Graduate School of Medicine, Ube, Japan; 2 Division of Laboratory, Yamaguchi University Hospital, Ube, Japan; 3 Division of Epidemiology and Prevention, Aichi Cancer Center Research Institute, Nagoya, Japan; 4 Department of Oncology and Laboratory Medicine, Yamaguchi University Graduate School of Medicine, Ube, Japan; 5 Department of Immunology, Yamaguchi University Graduate School of Medicine, Ube, Japan; 6 Department of Hematology and Oncology, Institute of Medical Science, The University of Tokyo, Tokyo, Japan; 7 Research Organization for Nano & Life Innovation, Waseda University, Tokyo, Japan

## Abstract

HLA matching is a well-known genetic requirement for successful bone marrow transplantation (BMT). However, the importance of non-HLA single-nucleotide polymorphisms (SNPs) remains poorly understood. The NLR family pyrin domain–containing 3 (*NLRP3*) inflammasome, a key regulator of innate immunity, is associated with multiple diseases. We retrospectively genotyped SNPs of *NLRP1–3* and caspase recruitment domain family member 8 (*CARD8*), which are implicated in the interleukin 1β (IL-1β) signaling, in 999 unrelated BMT donor–recipient pairs. We identified an association of the interaction between the recipient *NLRP3* SNP CC genotype and total HLA mismatches with grade 2–4 acute graft-versus-host disease (AGVHD), and an association of the interaction between the donor *NLRP3* SNP T allele and HLA-C mismatch with extensive chronic GVHD (ECGVHD), in both adjusted and unadjusted regressions (*P* < 0.005). Importantly, the ECGVHD risk associated with HLA-C mismatch was not elevated when the donor *NLRP3* genotype was CC. We also identified an association of the interaction between recipient *NLRP3* SNP and donor cytomegalovirus seropositivity with overall survival in adjusted regressions (*P* < 0.005). These results suggest the importance of certain SNP–covariate interactions in unrelated BMT. The three identified interactions may be useful for donor selection or outcome prediction.

## Introduction

Allogeneic hematopoietic stem cell transplantation (HSCT) can be classified according to donor relatedness and HSC source. In recent unrelated bone marrow transplantations (BMTs), HLA-A, -B, and -DRB1 were usually matched, whereas HLA-C remained mismatched in 15–30% of pairs^1^. HLA mismatches (MMs) are risk factors for mortality and graft-versus-host disease (GVHD)^2–4^.

Studies of non-HLA polymorphisms aimed at improving predictions of HSCT outcomes have produced conflicting results^5–10^, implying the existence of systematic confounding factors or interactions. Our group previously examined the relationship between a single-nucleotide polymorphism (SNP) in the nucleotide binding oligomerization domain containing 2 (*NOD2*) gene with acute GVHD (AGVHD), but found no significant association^11^. Another important player in innate immunity is the NLR family pyrin domain containing 3 (NLRP3) inflammasome, which senses danger signals and activates IL-1β and/or IL-18 signaling^12–16^. *NLRP3* was associated with relapse as a donor single-nucleotide polymorphism (SNP) in an HLA-identical sibling HSCT study of 133 Caucasian pairs^17^, but was later shown to promote AGVHD as a recipient gene in a murine BMT-based model^18–20^.

In this study, we sought to identify the associations between inflammasome SNPs and outcomes of unrelated BMT matched at least at HLA-A, -B, and -DRB1 from May 2006 to April 2009 through the Japan Marrow Donor Program (JMDP)^21^. We retrospectively genotyped two *NLRP3* SNPs and one SNP each from *NLRP1*, *NLRP2*, and caspase recruitment domain family member 8 (*CARD8*), which may also be involved in the IL-1β processing pathway^12,22–24^. In multivariable regressions, we tested not only a SNP of interest, but also the interactions between the SNP and the covariates retained through variable selection, that is, those interactions that are not only significant but also improve the Bayesian information criterion (BIC) of the model.

## Results

### Subjects and SNPs

The characteristics of the donors and patients are given in Supplementary Table S1. Among all 999 pairs, the median number of days before the final follow-up of the surviving recipients was 1090. The 822 malignant-disease patients without previous transplantation history (Group 1 in Supplementary Table S1) were used as subjects of main analyses. We will also describe the influence of excluding non-malignant disease patients without previous transplantation history and patients with previous transplantation history (Groups 2 and 3 in Supplementary Table S1) on major results. The outcomes analyzed for these 999 pairs are shown in Table 1. The five SNPs chosen for the *NLRP1–3* and *CARD8* genes are listed in Supplementary Table S2. These SNPs were successfully genotyped (Supplementary Table S3 and Supplementary Fig. S1). Allele frequencies were similar among the first-time transplantation recipients, donors, and 104 Japanese residents of Tokyo (JPT104) from the 1000 Genomes Project^25^, but the null hypothesis for Hardy–Weinberg equilibrium (HWE) was rejected for the recipient *NLRP1* SNP (Supplementary Table S4). We therefore excluded recipient *NLRP1* entirely from analysis. Linkage disequilibrium (LD) between the two *NLRP3* SNPs, intronic rs4612666 and downstream rs10925027, was similar among the donors, the recipients, and JPT104 (Supplementary Table S5).

**Table 1:** BMT outcomes of 999 donor–recipient pairs.

	Group 1* (N = 822)	Group 2* (N = 65)	Group 3* (N = 112)
AGVHD			
Grade 2–4	280	13	35
Grade 3–4	80	3	11
CGVHD			
All (limited + extensive)	235	16	25
Extensive	132	5	15
Death	354	12	65
Non-relapse mortality	182	12	33
Relapse	152	0	21
Neutrophil engraftment	782	62	103

^*^Groups 1, 2, and 3 are BMT pairs of malignant-disease patients without previous transplantation, non-malignant disease patients without previous transplantation, and patients who underwent previous transplantation, respectively. Competing events (see Methods) were taken into account. For example, CGVHD preceded by relapse was not counted as incidence of CGVHD.

### Grade 2–4 AGVHD

**Figure 1:**
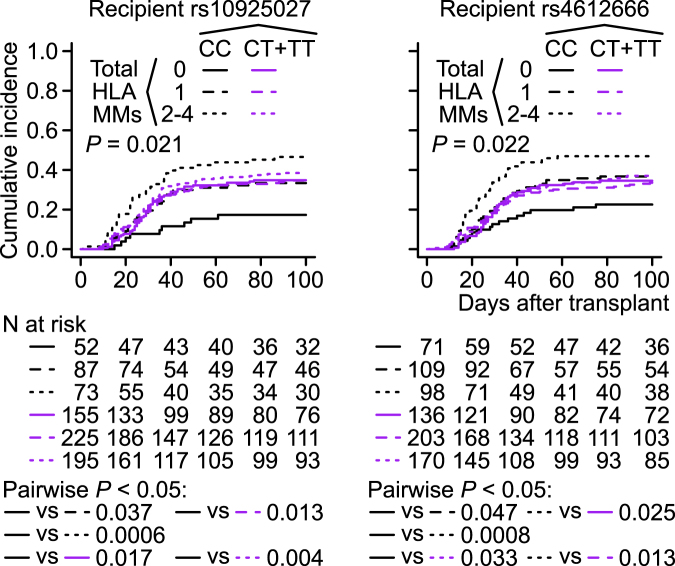
Unadjusted cumulative incidence curves (CICs) of grade 2–4 AGVHD according to the combinations between recipient *NLRP3* SNP genotypes and total HLA MMs. The malignant-disease first-time transplantation patients were included (N = 787). Excluded: AGVHD-unevaluable (N = 34) and day of grade 2/3/4 AGVHD unknown (N = 1). *P* values were determined by Gray’s test.

In univariable regression, no SNPs were significantly associated with grade 2–4 AGVHD (Supplementary Table S6). We analyzed grade 2–4 AGVHD by the directed multivariable regression fixing each SNP using a variable selection procedure (Methods). This procedure also tested for the presence of interactions between the SNP of interest and the other covariates retained after variable selection (i.e. cyclosporine A and total HLA MMs). Unexpectedly, the interaction between the recipient *NLRP3* SNP rs10925027 under the C-recessive model and total HLA MMs was retained through variable selection and was statistically significant (*P* = 0.002) (Table 2). The other recipient *NLRP3* SNP, rs4612666, also exhibited a considerable interaction (*P* = 0.010) (Table 2). The recipient rs10925027 interaction remained significant in multivariable regressions adjusted for reported risk factors of grade 2–4 or grade 3–4 AGVHD (i.e. cyclosporine A, recipient BMI, conditioning regimen, disease stage, donor age, recipient age, and female donor–male recipient)^4,26–28^, and also in unadjusted regression (Supplementary Table S7). The recipient rs4612666 interaction became significant when patients with non-malignant diseases were included (*P* = 0.004), whereas the recipient rs10925027 interaction remained significant in all patients (*P* < 0.001) (Supplementary Table S8). We plotted cumulative incidence curves (CICs) according to the six combinations between the HLA matching and the recipient *NLRP3* genotypes. Total HLA MMs were associated with an increase in AGVHD incidence only in the CC genotypes of these two recipient *NLRP3* SNPs (Fig. 1). The recipient *NLRP3* CC genotypes under at least two HLA MMs were associated with increased grade 2–4 AGVHD especially at earlier times, whereas the CC genotypes under the HLA 8/8 match were associated with a reduced risk of grade 2–4 AGVHD (Fig. 1).

**Table 2:** Multivariable subdistribution hazard (SH) regressions of grade 2–4 AGVHD, fixing total HLA MMs, a recipient *NLRP3* SNP, and their product interaction term.

	**rs10925027**		**rs4612666**	
	**SHR (95% CI)**	***P***	**SHR (95% CI)**	***P***
Total HLA MMs^*^	1.06 (0.92–1.23)	0.432	1.04 (0.89–1.22)	0.631
Recipient *NLRP3* SNP, C-recessive	0.55 (0.35–0.87)	0.010	0.71 (0.47–1.07)	0.102
Total HLA MMs × Rp *NLRP3* SNP Cr†	1.50 (1.15–1.94)	**0.002**	1.38 (1.08–1.77)	0.010
Cyclosporine A‡	1.43 (1.12–1.83)	**0.004**	1.41 (1.10–1.80)	0.007
	*P.xt* = 0.960 (df = 4)		*P.xt* = 0.369 (df = 4)	

*Total HLA MMs denote the sum of the numbers of MMs at HLA-C, -DQB1 and -DPB1 (HLA-A, -B and -DRB1 are matched). †‘Rp *NLRP3* SNP Cr’ stands for recipient *NLRP3* SNP under the C-recessive model (CC vs CT + TT), which refers to rs10925027 and rs4612666 in the left and right models, respectively. The symbol ‘ × ’ denotes the product interaction term between the two variables preceding and following it. ‡Yes vs no + unknown (see the legend of Supplementary Table S1 for details). SHR, subdistribution hazard ratio; CI, confidence interval; *P.xt*, *P* for the interaction between a variable and time; df, degrees of freedom. Only malignant-disease patients without previous transplantation history (Group 1 in Supplementary Table S1) were included (N = 787). Excluded: AGVHD-unevaluable (N = 34) and day of grade 2/3/4 AGVHD unknown (N = 1). The number of primary competing events (grade 2–4 AGVHD) = 280. *P* and *P.xt* were obtained by the Wald test. *P* < 0.005 is indicated in bold letters. The interaction term between total HLA MMs and recipient rs10925027 (in the left model) was retained throughout BIC-based variable selection (without fixation), when the three non-interaction terms were fixed.

### Extensive chronic GVHD (ECGVHD)

In univariable regression, no SNPs were significantly associated with ECGVHD (Supplementary Table S9). Unexpectedly, in the directed multivariable regression analysis, which also tested for the presence of an interaction between the SNP of interest and each of the covariates retained after variable selection (i.e. recipient BMI and HLA-C MM), the interaction between HLA-C MM and the donor *NLRP3* rs10925027 T allele was retained and significant (*P* = 0.002), and the other donor *NLRP3* SNP, rs4612666, exhibited a similar trend (*P* = 0.053) (Table 3). This significance was not the result of exclusion of patients with non-malignant diseases and/or previous transplantation history (Supplementary Table S10). The donor rs10925027 interaction remained significant in multivariable regressions adjusted for reported risk factors of both overall and extensive CGVHD (i.e. BMI, conditioning regimen, donor age, recipient age, and female donor–male recipient)^26,28,29^ in addition to disease stage, and also in unadjusted regression (Supplementary Table S11).

**Table 3:** Multivariable SH regressions of ECGVHD, fixing HLA-C MM, a donor *NLRP3* SNP, and their product interaction term.

	**rs10925027**		**rs4612666**	
	**SHR (95% CI)**	***P***	**SHR (95% CI)**	***P***
HLA-C MM	0.89 (0.47–1.70)	0.723	1.28 (0.75–2.17)	0.365
Donor *NLRP3* SNP, T-additive	1.02 (0.76–1.36)	0.914	1.10 (0.81–1.48)	0.552
HLA-C MM × Dn *NLRP3* SNP Ta*	2.02 (1.30–3.13)	**0.002**	1.50 (1.00–2.26)	0.053
Recipient BMI^†^	1.76 (1.25–2.47)	**0.001**	1.78 (1.27–2.51)	**<0.001**
	*P.xt* = 0.460 (df = 4)		*P.xt* = 0.919 (df = 4)	

^*^‘Dn *NLRP3* SNP Ta’ stands for donor *NLRP3* SNP under the T-additive model (TT vs CT vs CC), which refers to rs10925027 and rs4612666 in the left and right models, respectively. †High vs low + unknown (see Supplementary Table S1 for details). Only malignant-disease patients without previous transplantation history were included (N = 677). Excluded: CGVHD-unevaluable (N = 142) and day of CGVHD unknown (N = 3). The number of primary competing events (ECGVHD) = 132. The interaction term shown in the rs10925027 model was retained throughout BIC-based variable selection (without fixation), when the three non-interaction terms were fixed. The BICs of the left and right models were 1673 and 1679, respectively, and this BIC of the left model was superior to that of the lowest-BIC no-interaction model, which only retained HLA-C MM and recipient BMI with BIC = 1674. *P* and *P.xt* were obtained by the Wald test. See the legend of Table 2 for other notations.

**Figure 2:**
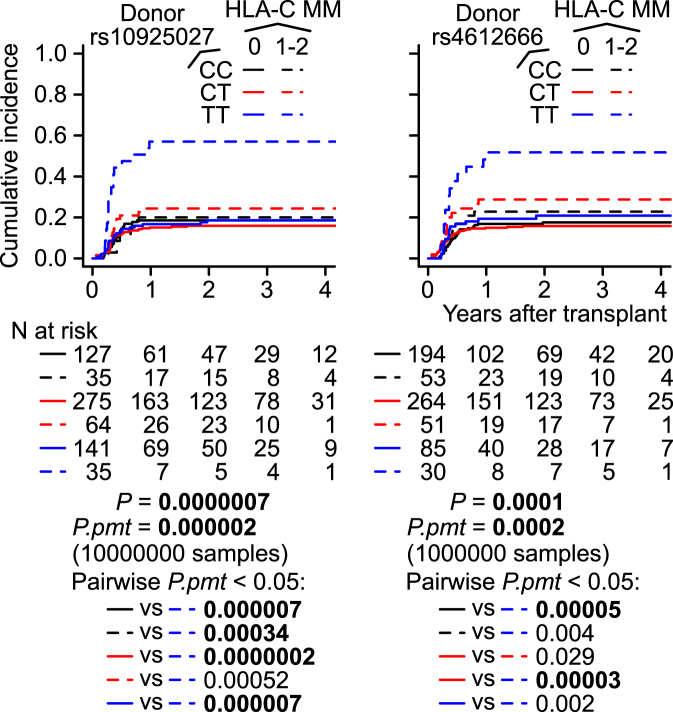
Unadjusted CICs of ECGVHD according to the combinations between donor *NLRP3* SNP genotypes and HLA-C MM. The malignant-disease first-time transplantation patients were included (N = 677). Excluded: CGVHD-unevaluable (N = 142) and day of CGVHD unknown (N = 3). *P* values were determined by Gray’s test. *P.pmt* stands for *P* determined by the sampled permutation-based Gray’s test.

Consistent with the results of these regressions, a CIC analysis showed that the HLA-C–mismatched donor *NLRP3* CT and TT genotypes exhibited slightly and sharply elevated incidences, respectively, and that the HLA-C–mismatched donor *NLRP3* CC genotype had an incidence approximately as low as those of the HLA 8/8-matched donors (Fig. 2).

### Overall survival (OS)

In univariable regression, no SNPs were significantly associated with OS (Supplementary Table S12). In the directed multivariable regression analysis, which also tested for the presence of an interaction between the SNP of interest and each of the covariates retained after variable selection (i.e. recipient age, performance status, disease stage, and donor CMV serostatus), the interaction between recipient *NLRP3* rs4612666 under the C-recessive mode and donor CMV serostatus was retained and significantly associated with OS (Supplementary Table S13). In this analysis, however, unknown status was merged with positive status, as for donor CMV. Therefore, we removed the 15 pairs with unknown donor CMV serostatus and repeated the regression for the two recipient *NLRP3* SNPs. The interaction between recipient *NLRP3* rs4612666 under the C-recessive mode and donor CMV serostatus was again retained and significantly associated with OS (*P* = 0.004) (Table 4). Furthermore, the interaction between recipient *NLRP3* rs10925027 under the C-additive model and donor CMV serostatus was also retained and significantly associated with OS (*P* = 0.005) (Table 5). The main-effect (non-interaction) term for recipient *NLRP3* rs10925027, which represents the effect of this SNP in patients transplanted from CMV-negative donors, was also significant (*P* = 0.001) (Table 5). Even when patients with non-malignant diseases and/or previous transplantation history were included, these interaction terms and non-interaction recipient *NLRP3* SNP terms were significant (Supplementary Table S14). However, donor CMV status and recipient CMV status were positively associated with each other (Supplementary Table S15). It is therefore possible that recipient CMV in addition to donor CMV is also involved in the interaction between recipient *NLRP3* and CMV, and that recipient CMV was not detected due to the smaller numbers of CMV-negative recipients relative to CMV-negative donors, as well as the positive correlation between donor and recipient CMV statuses. The number of donor–recipient CMV double-negative pairs was too small to perform an analysis of higher-order interactions (Supplementary Table S15). We also performed multivariable regression adjusted also with other reported risk factors of OS (i.e. donor age, ABO match, HLA-C mismatch)^4,27^, as well as unadjusted regression with these interactions fixed (Supplementary Tables S16 and S17). For both of the two recipient *NLRP3* SNPs, the interactions with donor CMV serostatus were significant in multivariable regressions adjusted with these reported risk factors (*P* = 0.004 and *P* = 0.004), but not in unadjusted regressions (*P* = 0.011 and *P* = 0.013).

**Table 4:** Multivariable Cox regression of OS fixing recipient *NLRP3* rs4612666, excluding patients with unknown donor CMV serostatus.

	**HR (95% CI)**	***P***
Donor CMV serostatus, positive vs negative	1.15 (0.86–1.55)	0.343
Recipient *NLRP3* rs4612666, C-recessive (CC vs CT + TT)	0.52 (0.32–0.83)	0.007
Donor CMV × recipient *NLRP3* rs4612666, C-recessive	2.21 (1.29–3.80)	**0.004**
Disease stage, advanced + unknown vs standard	1.76 (1.41–2.19)	**<0.001**
Recipient age, high vs low	1.72 (1.37–2.15)	**<0.001**
Recipient performance status, high vs low	1.49 (1.20–1.86)	**<0.001**
	*P.xt* = 0.014 (df = 6)	

Only malignant-disease patients without previous transplantation history (Group 1 in Supplementary Table S1) were included (N = 807). Excluded: Donor CMV serostatus unknown (*N* = 15). The number of events (death) = 346. *P* and *P.xt* were obtained by the Wald test. The interaction term between donor CMV serostatus and recipient rs4612666 (the third term) was retained throughout BIC-based variable selection (without fixation), when the non-interaction terms were fixed. HR, hazard ratio. See the legend of Table 2 for other notations.

**Table 5:** Multivariable Cox regression of OS fixing recipient *NLRP3* rs4612666, without patients with unknown donor CMV serostatus.

	**HR (95% CI)**	***P***
Donor CMV serostatus, positive vs negative	0.91 (0.60–1.37)	0.635
Recipient *NLRP3* rs10925027, C-additive (CC vs CT vs TT)	0.60 (0.44–0.81)	**0.001**
Donor CMV × recipient *NLRP3* rs10925027, C-additive	1.66 (1.17–2.36)	**0.0048**
Disease stage, advanced + unknown vs standard	1.76 (1.41–2.20)	**<0.001**
Recipient age, high vs low	1.74 (1.39–2.18)	**<0.001**
Recipient performance status, high vs low	1.47 (1.18–1.83)	**<0.001**
	*P.xt* = 0.253 (df = 6)	

Only malignant-disease patients without previous transplantation history (Group 1 in Supplementary Table S1) were analyzed (N = 807). Excluded: Donor CMV serostatus unknown (*N* = 15). The number of events (death) = 346. *P* and *P.xt* were obtained by the Wald test. The interaction term between donor CMV serostatus and recipient rs10925027 (the third term) was retained throughout BIC-based variable selection (without fixation), when the non-interaction terms were fixed. Note that the second term, which was also significant (*P* = 0.001), represents the effect of recipient rs10925027 in patients transplanted from CMV-negative donors, because CMV-positive and -negative statuses were coded as 1 and 0, respectively, in the model.

**Figure 3:**
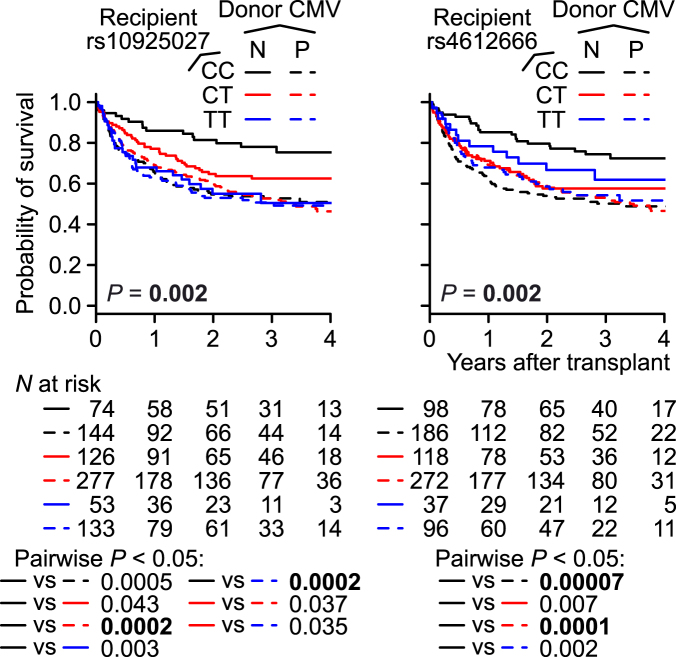
Unadjusted Kaplan–Meier survival curves (KMCs) of OS, according to the combinations between recipient *NLRP3* SNP genotypes and donor CMV serostatus. The malignant-disease first-time transplantation patients were included (N = 807). Excluded: donor CMV serostatus unknown (N = 15). Donor CMV serostatus is either negative (N) or positive (P). *P* values were determined by log-rank test.

Finally, we plotted Kaplan–Meier survival curves (KMCs) according to the six combinations between the recipient *NLRP3* SNP genotypes and donor CMV statuses (Fig. 3). Consistent with the regression analysis, the recipients who received transplants from CMV-negative donors exhibited the highest OS when their recipient *NLRP3* SNP genotype was CC. By contrast, in recipients who received transplants from CMV-positive donors, recipient *NLRP3* genotype was not visibly associated with OS, and the recipients who received transplants from CMV-positive donors exhibited, on average, worse OS in comparison with recipients who received transplants from CMV-negative donors, as expected from positive CMV serostatus being a known risk factor for OS^27,30^.

### Other outcomes

No SNPs were significantly associated with grade 3–4 AGVHD, overall CGVHD, engraftment, non-relapse mortality, or relapse with statistical significance (Supplementary Tables S18–S22).

## Discussion

In this study, we identified three interactions involving *NLRP3* SNPs associated with outcomes of unrelated BMT: an interaction between recipient *NLRP3* and total HLA MMs with grade 2–4 AGVHD; an interaction between donor *NLRP3* and HLA-C MM with ECGVHD; and an interaction between recipient *NLRP3* and donor CMV serostatus with OS. Possible mechanistic explanations for these associations could be inferred based on known functional consequences of these *NLRP3* SNPs: the C allele of the functional *NLRP3* SNP rs4612666 is expressed at higher levels than the other allele (T)^31^; Although the function of the other *NLRP3* SNP rs10925027 is unknown, rs10925027 is in LD at r^2^ = 0.64 with another functional *NLRP3* SNP, rs10754558, in JPT104 of 1000 Genomes Project, such that the G allele of rs10754558, which is the higher-expressed allele^31^, and the C allele of rs10925027 co-occur (Supplementary Tables S2 and S23). Hence, the C allele of the *NLRP3* SNP rs10925027 is also associated with the higher-expression allele. Thus, the C allele and the CC genotype should in theory represent the higher-expression allele and the highest-expression genotype, respectively, for both of the *NLRP3* SNPs chosen, rs4612666 and rs10925027.

According to these molecular functions, the CICs shown in Fig. 1 suggest that the putative highest-expression recipient *NLRP3* SNP genotype (CC) promotes grade 2–4 AGVHD, especially at earlier times, when at least two HLAs are mismatched. This result is remarkably consistent with the partial rescue/delay of AGVHD observed in *Nlrp3* ^−/−^ recipient mice that have undergone major histocompatibility complex-mismatched BMT^18^, and the synergy between total HLA MMs and the *NLRP3* high-expression genotype may be due at least in part to alloantigen-mediated T-cell proliferation mediated by recipient *NLRP3* ^18^. Many functional studies of immune-related genes in mice have used MHC-mismatched BMT models, whereas many of the human SNP studies for HSCT have used HLA highly-matched pairs. This is likely to be one of the reasons why the results of murine studies and human SNP studies have often been inconsistent.

By contrast, the CC genotype in the HLA 12/12-matched pairs was associated with a reduced incidence of grade 2–4 AGVHD (black straight lines in Fig. 1). Given that uric acid activates the murine NLRP3 inflammasome as a damage-associated molecular pattern^18^, this result is consistent with the reported association between low levels of uric acid and grade 2–4 AGVHD in HLA 10/10–matched HSCT^32^.

CGVHD is a poorly characterized complex disease^33,34^. We observed strong associations between ECGVHD and the lower-expression (T) alleles of the donor *NLRP3* SNPs under HLA-C MM, which may lead to decreased IL-1β (Table 3 and Fig. 2). To our knowledge, this is the first study in humans or animals to report the involvement of *NLRP3* in CGVHD, and at present there is no clear mechanistic explanation for the synergy between HLA-C MM and donor *NLRP3*. An interaction between a SNP and an HLA MM may represent a genetic interaction, which can occur either within the same pathway or between compensatory pathways^35^. Therefore, we cannot exclude the possibility that HLA-C MM and donor *NLRP3* act in parallel pathways. The involvement of HLA-C MM, as opposed to total HLA MMs, in this association of this interaction with ECGVHD appears to be consistent with a larger JMDP study of unrelated BMT, in which HLA-C MM was the only HLA-MM significantly associated with CGVHD^4^. The increase in ECGVHD due to the lower-expression *NLRP3* allele appears to be consistent with a recent study describing the roles of *NLRP3* in CD4^+^ T cells^36^ or with an IL-1β–independent role for *NLRP3* as a transcriptional regulator^37^. Regardless of the mechanisms, these results suggest opposing effects of recipient *NLRP3* on grade 2–4 AGVHD and donor *NLRP3* on ECGVHD in HLA-C mismatched pairs. These opposite actions, as well as the opposite effects of recipient *NLRP3* on grade 2–4 AGVHD between HLA-matched and -mismatched pairs, may need to be taken into account in future studies of *NLRP3* and the cytokines activated by it, namely IL-1β and IL-18.

We observed associations of better OS with the higher-expression (C) allele and the putative highest-expression genotype (CC) of the recipient *NLRP3* rs10920527 and rs4612666, respectively, only in the patients transplanted from the CMV-negative donors (Fig. 3). These results should be taken with caution, because the interactions between these recipient *NLRP3* SNPs and donor CMV status were statistically significant only in adjusted regressions (Supplementary Tables S15 and S16). CMV seropositivity, in donor or recipient, is a risk factor for OS even in recent HSCTs^30,38^.

Mouse CMV activates the AIM2 inflammasome, whereas the NLRP3 inflammasome is activated by RNA viruses and some other DNA viruses^13^. Therefore, the NLRP3 inflammasome is unlikely to play a direct role in a response to CMV. This notion is consistent with our observation that recipient *NLRP3* genotypes were not clearly associated with OS among recipients who received transplants from CMV-positive donors, assuming that the effect of CMV is dominant over that of the recipient *NLRP3* (broken lines in Fig. 3). The mechanism underlying the association of the higher-expression allele/genotype of the recipient *NLRP3* with better OS in the patients who received transplants from CMV-negative donors remains unclear, largely because these interactions were not significantly associated with NRM, relapse, or GVHD, but were probably derived from effects on both NRM and relapse.

This study has limitations. The first is its retrospective design. In particular, recipient SNPs run the risk of selection bias prior to BMT, which may have been reflected in the violation of HWE for recipient *NLRP1* rs11651270. It should be emphasized that clinical decisions should be based on well-controlled prospective studies. The NIH criteria for CGVHD diagnosis^39^ were not used in this study because the transplant registry for this study used the classical criteria for CGVHD^40^. Moreover, because allele-level HLA MM itself is an interaction, a SNP–HLA MM interaction is potentially a third-order interaction. Therefore it will be important to perform additional larger studies that take into account the HLA alleles of the recipient and corresponding donor. The same is true for interactions between a SNP and CMV status, for which both donor and recipient CMV statuses should be used simultaneously as covariates.

If validated, the interactions identified in this study may be useful in donor selection or outcome prediction. The cumulative incidence of ECGVHD in recipients who received transplants from donors with HLA-C MM and the highest-expression *NLRP3* genotype (CC) does not appear to substantially differ from that in recipients receiving transplants from HLA 8/8-matched donors (Fig. 2). Therefore, in cases in which there are several HLA-C mismatched donor candidates, a donor with the *NLRP3* CC genotype may be preferred in order to minimize the risk of ECGVHD. Likewise, the risk of grade 2–4 AGVHD may be higher for a recipient with the highest-expression *NLRP3* genotype (CC) and more than one HLA MM (Fig. 1). For a recipient with the highest-expression *NLRP3* genotype (CC), a CMV-negative donor may lead to better survival.

### Subjects, materials, and methods

#### Subjects

The subjects of this study were 999 donor–recipient pairs who satisfied all of the following criteria: the pair underwent an unrelated BMT matched at least at HLA-A, -B, and -DRB1 from May 2006 to April 2009 through the Japan Marrow Donor Program (JMDP)^21^; Japanese ethnicity; recipient days of survival were available; donor age was at least 20; HLA-A, -B, -C, -DRB1, -DQB1, and -DRB1 alleles were retyped and confirmed to be matched at HLA-A, -B, and -DRB1 (Supplementary Table S1). A recipient and the corresponding donor were either both included or both excluded. The final survey of clinical data was finished by September 2012 as described^4^. This study was conducted in accordance with the Declaration of Helsinki, and was approved by the institutional review boards of Yamaguchi University School of Medicine, the Institute of Medical Science of The University of Tokyo, and the JMDP. Written informed consent was obtained from all donors and recipients, and/or their legal guardians. No tissues were procured from prisoners. Some of the genotype and clinical data are available at the Japanese Genotype-phenotype Archive (JGA) under accession JGAS00000000071.

#### SNP selection

We considered both known functional SNPs with minor allele frequency > 0.1 in 104 (originally 89) Japanese residents of Tokyo (JPT104) from the 1000 Genomes Project^25^ and SNPs previously studied in the HSCT field. The chosen SNPs are listed in Supplementary Table S2. Known functional consequences and disease associations of these SNPs are detailed in Supplementary Methods.

#### SNP genotyping

Genomic DNA was purified from 200 µL of peripheral blood from each donor and recipient using the QIAamp DNA Blood Mini kit (Qiagen), and amplified using the Illustra GenomiPhi HY kit (GE Healthcare). SNP genotyping was carried out using TaqMan Genotyping Master Mix and TaqMan SNP Genotyping Assays (Applied Biosystems), listed in Supplementary Table S2, in a total volume of 5 µl using 20 ng of DNA in 384-well format on a 7900HT and/or ViiA 7 real-time PCR system (Applied Biosystems). Genotype calling was carried out using the software accompanying these systems. Only signals that passed the default threshold (quality = 95) were considered to be successfully genotyped. The un-genotyped samples and some of the successfully genotyped samples were genotyped by PCR, followed by direct Sanger sequencing, as detailed in Supplementary Methods.

#### Outcomes

Primary outcome was grade 2–4 AGVHD within 100 days after transplantation. Secondary outcomes were overall survival (OS), chronic GVHD (CGVHD), extensive CGVHD (ECGVHD), grade 3–4 AGVHD within 100 days, neutrophil engraftment, relapse, and non-relapse mortality (NRM). The (primary competing) events for OS and NRM were defined as death due to any cause and death without prior relapse, respectively. Relapse was defined as being positive for at least one clinical/hematological, cytogenetic, or molecular diagnosis. Neutrophil engraftment was defined as described^4^. AGVHD was graded by classical criteria^41,42^. CGVHD was diagnosed according to the Seattle criteria^43^. The day of CGVHD incidence was not necessarily after 100 days. Patients who were unevaluable for A/CGVHD (Supplementary Fig. S2) were excluded from the respective analyses. Diagnoses regarding GVHD, including day of incidence and unevaluable status, were the judgments of individual physicians. The time-to-event variables were defined as recently summarized^40^. The competing events for relapse and NRM were NRM and relapse, respectively. Those patients who had undergone BMT at an advanced stage and never achieved complete remission (CR) afterward (Supplementary Fig. S2) were excluded from analyses of relapse and NRM, as suggested for relapse-free survival^44^, and were treated as a competing event occurring on the day after BMT in all analyses of GVHD. Thus, the competing events for GVHD were relapse, death without prior occurrence of the corresponding GVHD or relapse, and lack of CR achievement.

#### Covariates

Disease stage was defined only for malignant disease patients, in which standard stage refers to chronic phase for CML and complete remission for the other malignant diseases. “Advanced” refers to stage other than the standard stage. Stage was set as “unknown” for solid tumor patients and patients whose stage data were missing. The GVH (graft-versus-host) and HVG (host-versus-graft) directions of the HLA MMs were defined at the allele level as described^4^. The effects of HLA MM on GVHD, NRM, relapse, and OS were examined in the GVH direction, whereas the effect on neutrophil engraftment was examined in the HVG direction. Myeloablative conditioning regimens were defined to exclude the reduced-intensity conditioning regimens^45^, and any regimens that included >5 Gy (>8 Gy if fractionated) total body irradiation, >9 mg/kg oral (>7.2 mg/kg if intravenously administered) busulfan, ≥140 mg/m^2^ of melphalan, or ≥10 mg/kg thiotepa.

### Statistical analysis

Phased linkage disequilibrium (LD) among the SNPs in JPT104 was calculated using VCFtools^46^ (ver. 0.1.11). Other analyses were performed in the R statistical environment (ver. 3.2.2) using the following packages: *genetics* (ver. 1.3) was used to assess the HWE and to calculate unphased LDs in the subjects and in JPT104; *survival* (ver. 2.38) was used to draw Kaplan–Meier survival curves (KMCs) and cumulative incidence curves (CICs), and for implementing the log-rank test and the Cox proportional hazard (PH) regression; *cmprsk* (ver. 2.2) was used for competing risk analyses, including Gray’s test and proportional sub-distribution hazard (SH) regression; *MASS* (ver. 7.3) and *crrstep* (ver. 2015–2) were used for variable selection in PH and SH regressions, respectively; *aod* (ver. 1.3) was used to perform the Wald test; *VennDiagram* (ver. 1.6) was used to draw a Venn diagram.

The sampled permutation log-rank and Gray’s chi-square tests were performed in a manner similar to the *permlogrank* function in the *clinfun* package (ver. 1.0): the variables of interest were permuted without replacement with respect to the rest of data, after which the chi-square test statistics for overall comparison, as well as pairwise comparison, were calculated from the same permuted data. This permutation procedure was repeated 10^5^, 10^6^, or 10^7^ times at random, after which the proportion of resampled chi-square statistics greater than or equal to the original values were calculated as *P.pmt*. The times used in the log-rank and Gray’s tests were always the number of days until the maximum follow-up period, regardless of the years displayed in KMCs/CICs. It should be noted that the overall significant difference in the HLA MM–SNP combinations, or the lack thereof, does not refer to the presence or absence of an interaction (e.g., a difference simply between HLA matched vs. mismatched may lead to such significance), and that the presence of an interaction is assessed by direct tests of the interaction terms in regressions.

In regression analyses, all categorical and binary-converted variables were entered as 1 or 0 as described^47^, except that the individual HLA MMs and the SNPs under the additive model could take the value of 0, 1, or 2. These 0/1/2 values and their product interaction terms were generated as data, but not through the regression formula. To facilitate and simplify multivariable analyses, missing values for covariate data were merged into one subcategory, as described in the Legend of Supplementary Table S1, unless stated otherwise. It should be noted that the dominant model for one of the two alleles of each SNP is equivalent to the recessive model for the other allele with a reciprocal coding scheme, and hence (S)HR. For example, the T-dominant model for rs10925027 is coded as CT/TT = 1 and CC = 0, whereas the C-recessive model is as CT/TT = 0 and CC = 1. Likewise, the T-additive model for this SNP was coded as CC = 0, CT = 1, and TT = 2, whereas the C-additive model was coded as TT = 0, CT = 1, and CC = 2. The PH and proportional SH assumptions in Cox and Fine–Gray models, respectively, were tested by introducing time–variable interaction term(s), which were examined by the Wald test to obtain *P.xt*, similar to a described method^47^.

In multivariable PH/SH regression analyses, the SNPs under the additive or minor allele–dominant models and the clinical variables shown in Supplementary Table S1 (other than UD and previous transplantation history) that exhibited *P* < 0.1 without violating the proportional assumption in univariable regression were subjected to backward variable elimination based on Bayesian information criterion (BIC). The individual HLA MMs and total HLA MMs were separately used in each selection process. In directed multivariable PH/SH regression analyses, the SNP of interest (and, in some cases, certain covariates) were fixed in the model, whereas other covariates were subjected to elimination. All the product interaction terms between the SNP of interest and the other retained covariates were introduced, and then subjected to another round of BIC-based backward selection, with the non-interaction terms fixed. If the estimates of (S)HRs for a SNP under the additive model, an HLA MM, and their product interaction term are A, B, and C, respectively, then the estimate of (S)HR for having U risk alleles and V MMs, where U and V are 0, 1, or 2, is given by A^U^ × B^V^ × C^U×V^. The estimates under the dominant/recessive model can be obtained similarly. All *P* values reported in this study are two-tailed unadjusted values. The significance level α = 0.005 for all tests in this study, except for the pairwise comparisons of KMC/CIC, for which this α-level is further corrected by the Holm–Bonferroni method using the number of combinations (i.e., 6 or 15).

## Electronic supplementary material

Supplementary Information
