# Supplementary material for: Associations of interactions between NLRP3 SNPs and HLA mismatch with acute and extensive chronic graft-versus-host diseases
Source: Sci Rep. 2017 Oct 12;7:13097. doi: 10.1038/s41598-017-13506-w (PMC5638959; doi:10.1038/s41598-017-13506-w)
Supplement: Supplementary file 1 — Supplementary Information [file 41598_2017_13506_MOESM1_ESM.pdf]

Supplementary Information for

Associations of interactions between *NLRP3* SNPs and HLA mismatch with acute and extensive chronic graft-versus-host diseases

Hidekazu Takahashi[1], Naoko Okayama[2], Natsu Yamaguchi[1], Yuta Miyahara[2], Yasuo Morishima[3], Yutaka Suehiro[4], Takahiro Yamasaki[2,4], Koji Tamada[5], Satoshi Takahashi[6], Arinobu Tojo[6], Shigetaka Asano[7] & Tsuyoshi Tanabe[1]

[1]Department of Public Health and Preventive Medicine, Yamaguchi University Graduate School of Medicine, Ube, Japan. [2]Division of Laboratory, Yamaguchi University Hospital, Ube, Japan. [3]Division of Epidemiology and Prevention, Aichi Cancer Center Research Institute, Nagoya, Japan. [4]Department of Oncology and Laboratory Medicine, Yamaguchi University Graduate School of Medicine, Ube, Japan. [5]Department of Immunology, Yamaguchi University Graduate School of Medicine, Ube, Japan. [6]Department of Hematology and Oncology, Institute of Medical Science, The University of Tokyo, Tokyo, Japan. [7]Research Organization for Nano & Life Innovation, Waseda University, Tokyo, Japan. Correspondence and requests for materials should be addressed to T.T. (e-mail: tanabe@yamaguchi-u.ac.jp)

Table of Contents

Content **Pages**

Table S1. Characteristics of 999 donor–recipient pairs who underwent unrelated BMT..........4

Table S2. SNP information..........5

Table S3. Summary of genotyping of 999 pairs (1998 subjects)..........6

Table S4. SNP frequency..........7

Table S5. Unphased linkage disequilibrium (LD) among the SNPs..........8

Table S6. Univariable subdistribution hazard (SH) regression of grade 2–4 AGVHD..........9

Table S7. SH Regressions of grade 2–4 AGVHD, fixing the interaction between total HLA MMs and recipient *NLRP3* rs10925027 under the C-recessive model, adjusted or unadjusted by reported risk factors..........10

Table S8 Multivariable regressions of grade 2–4 AGVHD in all-disease patients..........11

Table S9. Univariable SH regression of ECGVHD..........12

Table S10. Multivariable regressions of ECGVHD in all-disease patients..........13

Table S11. SH regressions of ECGVHD fixing the interaction between HLA-C MM and donor *NLRP3* rs10925027, adjusted or unadjusted by reported risk factors..........14

Table S12. Univariable Cox regression of overall survival..........15

Table S13. Directed multivariable Cox regression of OS fixing recipient *NLRP3* rs4612666...16

Table S14. Multivariable Cox regressions of OS in relation to the interaction between recipient *NLRP3* SNP and donor CMV serostatus in patients with all diseases..........17

Table S15. Association between donor and recipient CMV status..........19

Table S16. Cox regressions of OS fixing the interaction between recipient *NLRP3* rs4612666 and donor CMV serostatus, adjusted or unadjusted by reported risk factors..........20

Table S17. Cox regressions of OS fixing the interaction between recipient *NLRP3* rs10925027 and donor CMV serostatus, adjusted or unadjusted by reported risk factors..........21

Table S18. Univariable SH regression of grade 3–4 AGVHD..........22

Table S19. Univariable SH regression of overall CGVHD..........23

Table S20. Univariable SH regression of neutrophil engraftment..........24

Table S21. Univariable SH regression of non-relapse mortality (NRM) 25

Table S22. Univariable SH regression of relapse 26

Table S23. Known SNPs in linkage disequilibrium with the five SNPs analyzed 27

Figure S1. Representative genotyping results 28

Figure S2. Venn diagram of AGVHD-unevaluable status, CGVHD-unevaluable status, engraftment failure, and no achievement of complete remission after BMT 30

Supplementary Methods 31

Supplementary References 32

Table S1. Characteristics of 999 donor–recipient pairs who underwent unrelated BMT

Recipient characteristics	Group 1* (N = 822)	Group 2* (N = 65)	Group 3* (N = 112)
Sex			
Female	336	26	42
Male	486	39	70
Age, year			
Low (1-44)	379	54	61
High (45-71)	443	11	51
Underlying disease			
Malignant	*(822)*	(0)	*(110)*
Acute myeloid leukemia (AML)	342	0	38
Acute lymphoblastic leukemia (ALL)	178	0	17
Myelodysplastic syndromes (MDS)	120	0	3
Chronic myeloid leukemia (CML)	27	0	1
Other leukemia	66	0	0
Lymphoid malignancy (LM)	76	0	40
Myeloproliferative disorders (MPD)	8	0	1
Plasma cell dyscrasias (PCD)	3	0	10
Solid tumors (ST)	2	0	0
Non-malignant	*(0)*	*(65)*	*(2)*
Hematopoietic disorder†	0	48	1
Primary immunodeficiency	0	10	0
Epstein–Barr virus infection	0	4	0
Inborn error of metabolism	0	3	1
Disease stage			
Standard	450	N.A.	N.A.
Advanced‡	365	N.A.	N.A.
Unknown‡	7	N.A.	N.A.
Body mass index (BMI), kg/m^2			
Unknown‡	3	0	0
Low (12.0-22.0)‡	466	49	68
High (22.0-38.6)	353	16	44
CMV serostatus before BMT			
Negative	141	15	24
Positive‡	626	47	83
Unknown‡	55	3	5
Performance status (PS) before BMT			
Low (0)	478	35	59
High (1-4)	344	30	53
Previous transplantation history			
No	822	65	0
Yes	0	0	112

Donor characteristics	Group 1* (N = 822)	Group 2* (N = 65)	Group 3* (N = 112)
Sex			
Female	260	22	33
Male	562	43	79
Age			
Low (20-33 years)	396	38	56
High (34-68 years)	426	27	56
CMV serostatus before BMT			
Negative	253	15	26
Positive‡	554	50	82
Unknown‡	15	0	4

Transplantation	Group 1*	Group 2*	Group 3*
HSC source			
Bone marrow	822	65	112
Other	0	0	0
Myeloablative conditioning			
No	177	50	53
Yes	645	15	59
Cyclosporine A (CyA)§			
Unknown‡	1	0	0
No‡	596	55	87
Yes	225	10	25
Ara-C			
No	731	65	101
Yes	91	0	11
Cyclophosphamide			
No	354	12	82
Yes	468	53	30
N of nucleated cells infused¶			
Unknown‡	46	5	5
Low (0.01-2.50)‡	384	28	61
High (≥2.50)	392	32	46
Days from diagnosis to BMT			
Low (9-269)	415	10	N.A.
High (270-10897)‡	406	55	N.A.
Unknown‡	1	0	N.A.

Matching characteristics	Group 1* (N = 822)	Group 2* (N = 65)	Group 3* (N = 112)
ABO blood type			
Match	464	38	65
Mismatch	358	27	47
HLA-A, -B or -DRB1			
0 mismatches	822	65	112
≥1 mismatch (either direction)	0	0	0
HLA-C			
0 mismatches	636	50	91
1 mismatch (both directions)	147	14	17
1 mismatch (GVH direction)	9	0	1
1 mismatch (HVG direction)	16	0	2
2 mismatches (both directions)	14	1	1
HLA-DQB1			
0 mismatches	758	55	107
1 mismatch (both directions)	52	8	4
1 mismatch (GVH direction)	5	0	0
1 mismatch (HVG direction)	7	1	1
2 mismatches (both directions)	0	1	0
HLA-DPB1			
0 mismatches	195	21	26
1 mismatch (both directions)	331	19	47
1 mismatch (GVH direction)	68	11	6
1 mismatch (HVG direction)	68	3	7
2 mismatches (both directions)	160	11	26
HLA-C, -DQB1 or -DPB1, GvH direction			
0 mismatches	214	19	28
1 mismatch (GVH direction)	330	23	46
2 mismatches (GVH direction)	211	16	32
3 mismatches (GVH direction)	60	5	5
4 mismatches (GVH direction)	7	2	1
HLA-C, -DQB1 or -DPB1, bi-directional			
0 mismatches	155	16	20
1 mismatch (either direction)	369	26	52
2 mismatches (either direction)	223	15	34
3 mismatches (either direction)	64	6	5
4 mismatches (either direction)	11	2	1

CMV, cytomegalovirus; Ara-C, arabinofuranosyl cytidine; GVH, graft-versus-host; HVG, host-versus-graft; N.A., not applicable.
*Groups 1, 2, and 3 are BMT pairs of malignant-disease patients without previous transplantation, non-malignant disease patients without previous transplantation, and patients who underwent previous transplantation, respectively. †E.g., aplastic anemia. ‡These two subcategories are merged into one subcategory to enable/simplify multivariable analyses. §Tacrolimus was administered to 594 of the 596 CyA non-users and in 6 of the 225 CyA users in Group 1. ¶10^8 per kg body weight.

Table S2. SNP information

SNP	Alternative name	Gene	SNP type	Chromosome	Location in GRCh37*	Location in GRCh38*	Allele 1†	Allele 2†
rs11651270	M1184V	*NLRP1*	Non-synonymous	17	5425077	5521757	T(Met)	C(Val)
rs1043673	A1052E	*NLRP2*	Non-synonymous	19	55512232	55000864	C(Ala)	A(Glu)
rs4612666	-	*NLRP3*	Intron	1	247599070	247435768	C	T
rs10925027	-	*NLRP3/OR2B11*	Downstream	1	247612562	247449260	C	T
rs2043211	C10X	*CARD8*	Nonsense	19	48737706	48234449	A(Cys)	T(Stop)

SNP	Reported molecular function of allele 2 relative to allele 1	DNA sequence around the SNP
rs11651270	Higher autoproteolysis and higher IL-1β release[1]	CTTTAAAGTGGGCCA[**T/C**]TTGGAACAGGGATGT
rs1043673	-	AACATCATCCCTGGG[**C/A**]AGAAAGGCCTTCTTC
rs4612666	Lower enhancer activity and lower signal in electrophoretic mobility shift assay[2]	AGGGAGCTGGGAAGA[**C/T**]GTAGTATTGGTGGGA
rs10925027	-	ATGAGTGGAATTTCA[**C/T**]CTTTGATAGGATAGG
rs2043211	Nonsense substitution resulting in abrogation of inhibition of NF-κB activity[3]	CAGGAACAGCACGGA[**A/T**]CAATAATGGCTCTGC

SNP	TaqMan Assay ID	PCR primer A (5' -> 3') used for direct sequencing	PCR primer B (5' -> 3') used for direct sequencing
rs11651270	C__31558200_10	CCATGTGGACACATCCCTGTTC	CATTATGGATCATTTTCAGGAGGACTC
rs1043673	C___8717916_20	ATGATGAACTCAATAAGCTGCTGGAAG	CTGGAAAATCGATGACTTCATGGAG
rs4612666	C__26646029_10	ATGGTTGCACAACAATGTGAAGTG	ACAAGTAAGCATTCTCCAAGCTCC
rs10925027	C__30713882_10	GTCACTTTGGCAAATGATTCCATTGTAG	GCTCTGATACTCTCAAACCTTTCCCTC
rs2043211	C__11708080_1_	CTGTTGTTGTTTTCCAGGGTATACAGG	CTCTGTGATATTGAGACACAGCGTC

TaqMan assays and oligo DNA sequences used in this study are shown.

*GRCh37 and 38 stand for Genome Reference Consortium human genome (build 37 and 38), respectively.

†Alleles 1 and 2 exhibit variant nucleotides with putative amino-acid residues in parentheses. “Stop” refers to a nonsense codon.

Table S3. Summary of genotyping of 999 pairs (1998 subjects)

	Gene	N of subjects successfully genotyped in one round of the TaqMan assay	N of subjects successfully genotyped by PCR direct sequencing after a failure in the TaqMan assay	N of subjects genotyped only by PCR direct sequencing
rs10925027	*NLRP3/OR2B11*	1965	33	0
rs4612666	*NLRP3*	1960	28	10
rs2043211	*CARD8*	1954	34	10
rs1043673	*NLRP2*	1965	23	10
rs11651270	*NLRP1*	1920	70	8

Table S4. SNP frequency

	Donor		Recipient		1000 Genomes
	Malignant diseases	All diseases	Malignant diseases	All diseases	JPT104
N	822	887	822	887	104
rs10925027 (*NLRP3*)					
CC	197	214	223	245	30
CT	401	438	409	437	51
TT	224	235	190	205	23
T allele frequency	0.516	0.512	0.480	0.477	0.466
P for HWE	0.530	0.737	0.944	0.736	0.846
rs4612666 (*NLRP3*)					
CC	286	311	288	311	42
CT	394	424	398	433	43
TT	142	152	136	143	19
T allele frequency	0.412	0.410	0.408	0.405	0.389
P for HWE	0.773	0.729	1.000	0.728	0.215
rs2043211 (*CARD8*)					
AA	315	332	320	348	33
AT	386	425	367	398	51
TT	121	130	135	141	20
T allele frequency	0.382	0.386	0.387	0.383	0.438
P for HWE	0.883	0.777	0.091	0.136	1.000
rs1043673 (*NLRP2*)					
CC	496	535	490	526	68
AC	285	308	280	304	28
AA	41	44	52	57	8
A allele frequency	0.223	0.223	0.234	0.236	0.212
P for HWE	1.000	1.000	0.172	0.162	0.072
rs11651270 (*NLRP1*)					
TT	416	450	445	478	52
CT	336	363	296	323	46
CC	70	74	81	86	6
C allele frequency	0.290	0.288	0.279	0.279	0.279
P for HWE	0.865	0.935	**0.003**	0.006	0.463

Genotype frequency and the Hardy–Weinberg equilibrium (HWE) of the five SNPs in malignant-disease first-time transplantation recipients and their donors (Groups 1 in Supplementary Table S1), in all-disease first-time transplantation recipients and their donors (Groups 1+2 in Supplementary Table S1), and in Japanese residents of Tokyo (JPT104) of 1000 Genomes Project are shown. Bold letters indicate $P < 0.005$. Yellow or light green highlighting indicates $P < 0.05$ or $P < 0.01$, respectively (exact test).

Table S5. Unphased linkage disequilibrium (LD) among the SNPs

**Donor (N = 887)**	**rs10925027 (*NLRP3*)**	**rs4612666 (*NLRP3*)**	**rs2043211 (*CARD8*)**	**rs1043673 (*NLRP2*)**
**rs4612666 (*NLRP3*)**	0.28*			
**rs2043211 (*CARD8*)**	0.00	0.00		
**rs1043673 (*NLRP2*)**	0.00	0.00	0.00	
**rs11651270 (*NLRP1*)**	0.00	0.00	0.00	0.00

**Recipient (N = 887)**	**rs10925027 (*NLRP3*)**	**rs4612666 (*NLRP3*)**	**rs2043211 (*CARD8*)**	**rs1043673 (*NLRP2*)**
**rs4612666 (*NLRP3*)**	0.27*			
**rs2043211 (*CARD8*)**	0.00	0.00		
**rs1043673 (*NLRP2*)**	0.00	0.00	0.00	
**rs11651270 (*NLRP1*)**	0.00	0.00	0.00	0.00

**JPT104 (N = 104)**	**rs10925027 (*NLRP3*)**	**rs4612666 (*NLRP3*)**	**rs2043211 (*CARD8*)**	**rs1043673 (*NLRP2*)**
**rs4612666 (*NLRP3*)**	0.23*			
**rs2043211 (*CARD8*)**	0.00	0.01		
**rs1043673 (*NLRP2*)**	0.00	0.00	0.00	
**rs11651270 (*NLRP1*)**	0.01	0.02	0.02	0.00

The r^2 values among the SNPs in all-disease first-time transplantation recipients (Groups 1+2 in Supplementary Table S1), in their donors, and in JPT104 are shown. *$P < 0.005$ (chi-square test).

Table S6. Univariable subdistribution hazard (SH) regression of grade 2–4 AGVHD

Recipient SNPs

		Additive model				Dominant model				Recessive model			
**Gene**	**SNP**		**SHR (95% CI)**	***P***	***P.xt***		**SHR (95% CI)**	***P***	***P.xt***		**SHR (95% CI)**	***P***	***P.xt***
NLRP1	rs11651270*	N of the C allele	N.A.	N.A.	N.A.	CT+CC vs TT	N.A.	N.A.	N.A.	CC vs TT+CT	N.A.	N.A.	N.A.
NLRP2	rs1043673	N of the A allele	1.12 (0.93–1.35)	.230	.023	AC+AA vs CC	1.07 (0.84–1.35)	.585	.050	AA vs CC+AC	1.50 (1.01–2.22)	.043	.051
NLRP3	rs4612666	N of the T allele	0.98 (0.82–1.16)	.777	.110	CT+TT vs CC	0.92 (0.72–1.17)	.503	.061	TT vs CC+CT	1.05 (0.77–1.43)	.744	.611
NLRP3	rs10925027	N of the T allele	1.02 (0.87–1.20)	.784	.151	CT+TT vs CC	1.07 (0.81–1.39)	.642	.370	TT vs CC+CT	1.00 (0.76–1.31)	.972	.131
CARD8	rs2043211	N of the T allele	1.02 (0.87–1.21)	.799	.422	AT+TT vs AA	1.02 (0.81–1.30)	.856	.361	TT vs AA+AT	1.04 (0.76–1.43)	.804	.697

Donor SNPs

		Additive model				Dominant model				Recessive model			
**Gene**	**SNP**		**SHR (95% CI)**	***P***	***P.xt***		**SHR (95% CI)**	***P***	***P.xt***		**SHR (95% CI)**	***P***	***P.xt***
NLRP1	rs11651270	N of the C allele	0.96 (0.80–1.15)	.649	.809	CT+CC vs TT	0.98 (0.78–1.24)	.892	.663	CC vs TT+CT	0.84 (0.54–1.31)	.434	.825
NLRP2	rs1043673	N of the A allele	1.05 (0.86–1.28)	.644	.854	AC+AA vs CC	1.07 (0.85–1.36)	.573	.410	AA vs CC+AC	0.99 (0.56–1.77)	.984	.076
NLRP3	rs4612666	N of the T allele	0.96 (0.81–1.14)	.654	.679	CT+TT vs CC	0.96 (0.76–1.23)	.763	.876	TT vs CC+CT	0.93 (0.67–1.28)	.654	.340
NLRP3	rs10925027	N of the T allele	0.97 (0.82–1.13)	.669	.565	CT+TT vs CC	1.04 (0.79–1.37)	.790	.339	TT vs CC+CT	0.88 (0.67–1.15)	.353	.975
CARD8	rs2043211	N of the T allele	0.89 (0.75–1.06)	.183	.562	AT+TT vs AA	0.87 (0.68–1.10)	.246	.504	TT vs AA+AT	0.84 (0.60–1.18)	.315	.860

The results for each SNP were obtained by running separate regressions under the three genetic models indicated. Only malignant disease patients without previous transplantation history were analyzed (N = 787). Excluded: AGVHD-unevaluable (N = 34) and the day of grade 2/3/4 AGVHD unknown (N = 1). The number of the primary competing events (grade 2–4 AGVHD) = 280. SHR, subdistribution hazard ratio; CI, confidence interval; *P.xt*, *P* for the interaction between a variable and time. *P* and *P.xt* were obtained by the Wald test. Bold letters indicate $P < 0.005$ or $P.xt < 0.005$. Yellow highlighting indicates $P < 0.05$.

*Not applicable (N.A.) due to the violation of HWE.

Table S7. SH Regressions of grade 2–4 AGVHD, fixing the interaction between total HLA MMs and recipient *NLRP3* rs10925027 under the C-recessive model, adjusted or unadjusted by reported risk factors

	**Model 1**		**Model 2**	
	**SHR (95% CI)**	***P***	**SHR (95% CI)**	***P***
Total HLA MMs	1.07 (0.92–1.24)	.376	1.06 (0.92–1.23)	.401
Recipient *NLRP3* rs10925027, C-recessive	0.53 (0.33–0.84)	.007	0.56 (0.35–0.89)	.013
Total HLA MMs × Rp rs10925027 Cr*	1.51 (1.16–1.97)	**.002**	1.48 (1.14–1.94)	**.004**
Cyclosporine A, yes vs no+unknown	1.43 (1.12–1.83)	**.005**		
Recipient BMI, high vs low+unknown	0.85 (0.66–1.08)	.187		
Myeloablative, yes vs no	1.10 (0.81–1.50)	.531		
Disease stage†	1.27 (1.00–1.62)	.049		
Recipient age, high vs low	0.78 (0.61–1.01)	.056		
Donor age, high vs low	1.30 (1.02–1.65)	.033		
Female donor–male recipient‡	1.07 (0.79–1.46)	.644		
	P.xt = 0.925 (df = 10)		*P.xt* = 0.883 (df = 3)	

Model 1 includes reported risk factors for grade 2–4 or 3–4 AGVHD, in addition to the interaction between total HLA MMs and recipient *NLRP3* rs10925027. Model 2 shows the unadjusted regression according to total HLA MMs, recipient *NLRP3* rs10925027, and their interaction. Malignant-disease patients without previous transplantation history were included (N = 787). Excluded: AGVHD-unevaluable (N = 34) and day of grade 2/3/4 AGVHD unknown (N = 1). The number of primary competing events (grade 2–4 AGVHD) = 280. *P* and *P.xt* were obtained by the Wald test.

*'Rp rs10925027 Cr' stands for recipient *NLRP3* rs10925027 under the C-recessive model (CC vs CT+TT). †Advanced+unknown vs standard. ‡vs female donor–female recipient, male donor–male recipient, and male donor–female recipient, combined. See the legend of Table 2 for other notations.

Table S8 Multivariable regressions of grade 2–4 AGVHD in all-disease patients

All patients without previous transplantation history (Group 1+2 in Supplementary Table S1)

	rs10925027		rs4612666	
	**SHR (95% CI)**	***P***	**SHR (95% CI)**	***P***
Total HLA MMs	1.07 (0.93–1.23)	.374	1.04 (0.89–1.21)	.630
Recipient *NLRP3* SNP, C-recessive	0.54 (0.34–0.85)	.008	0.67 (0.45–1.01)	.055
Total HLA MMs × Rp *NLRP3* SNP*	1.51 (1.17–1.94)	**.002**	1.42 (1.12–1.81)	**.004**
Cyclosporine A†	1.43 (1.12–1.83)	**.004**	1.40 (1.10–1.79)	.007
	P.xt = 0.982 (df = 4)		*P.xt* = 0.420 (df = 4)	

All patients (Group 1+2+3 in Supplementary Table S1)

	rs10925027		rs4612666	
	**SHR (95% CI)**	***P***	**SHR (95% CI)**	***P***
Total HLA MMs	1.05 (0.92–1.20)	.486	1.04 (0.90–1.20)	.638
Recipient *NLRP3* SNP, C-recessive	0.52 (0.33–0.80)	**.003**	0.70 (0.48–1.03)	.072
Total HLA MMs × Rp *NLRP3* SNP*	1.53 (1.19–1.96)	**<.001**	1.38 (1.10–1.74)	.006
Cyclosporine A†	1.38 (1.09–1.73)	.007	1.35 (1.07–1.71)	.011
	P.xt = 0.953 (df = 4)		*P.xt* = 0.358 (df = 4)	

SH regressions were conducted fixing total HLA MMs, recipient *NLRP3* SNP, their interaction, and cyclosporine A. In the upper two models, all (including non-malignant)-disease first-time transplantation patients were included (N = 849); AGVHD-unevaluable (N = 37) and day of grade 2/3/4 AGVHD unknown (N = 1) were excluded. The number of primary competing events (grade 2–4 AGVHD) = 293. In the lower two models, all patients were included (N = 950); AGVHD-unevaluable (N = 48) and day of grade 2/3/4 AGVHD unknown (N = 1) were excluded. The number of primary competing events (grade 2–4 AGVHD) = 328. *P* and *P.xt* were obtained by the Wald test.

*'Rp *NLRP3* SNP' stands for recipient *NLRP3* SNP, referring to rs10925027 (left model) or rs4612666 (right model), under the C-recessive model (CC vs CT+TT). †Yes vs no+unknown. See the legend of Table 2 for other notations.

Table S9. Univariable SH regression of ECGVHD

Recipient SNPs

		Additive model				Dominant model				Recessive model			
**Gene**	**SNP**		**SHR (95% CI)**	***P***	***P.xt***		**SHR (95% CI)**	***P***	***P.xt***		**SHR (95% CI)**	***P***	***P.xt***
NLRP1	rs11651270*	N of the C allele	N.A.	N.A.	N.A.	CT+CC vs TT	N.A.	N.A.	N.A.	CC vs TT+CT	N.A.	N.A.	N.A.
NLRP2	rs1043673	N of the A allele	0.98 (0.72–1.32)	.884	.720	AC+AA vs CC	0.91 (0.64–1.28)	.580	.956	AA vs CC+AC	1.32 (0.67–2.61)	.421	.393
NLRP3	rs4612666	N of the T allele	1.08 (0.85–1.37)	.543	.054	CT+TT vs CC	1.07 (0.75–1.54)	.706	.031	TT vs CC+CT	1.16 (0.75–1.78)	.505	.402
NLRP3	rs10925027	N of the T allele	1.03 (0.81–1.33)	.789	.489	CT+TT vs CC	0.94 (0.64–1.37)	.751	.470	TT vs CC+CT	1.17 (0.79–1.73)	.421	.727
CARD8	rs2043211	N of the T allele	1.03 (0.80–1.31)	.839	.091	AT+TT vs AA	1.01 (0.72–1.43)	.946	.209	TT vs AA+AT	1.07 (0.67–1.71)	.763	.137

Donor SNPs

		Additive model				Dominant model				Recessive model			
**Gene**	**SNP**		**SHR (95% CI)**	***P***	***P.xt***		**SHR (95% CI)**	***P***	***P.xt***		**SHR (95% CI)**	***P***	***P.xt***
NLRP1	rs11651270	N of the C allele	0.76 (0.57–1.01)	.055	.370	CT+CC vs TT	0.74 (0.52–1.04)	.081	.445	CC vs TT+CT	0.61 (0.28–1.32)	.209	.580
NLRP2	rs1043673	N of the A allele	0.82 (0.60–1.12)	.205	.834	AC+AA vs CC	0.81 (0.57–1.15)	.242	.992	AA vs CC+AC	0.68 (0.25–1.85)	.449	.176
NLRP3	rs4612666	N of the T allele	1.26 (0.99–1.61)	.064	.679	CT+TT vs CC	1.16 (0.82–1.66)	.402	.215	TT vs CC+CT	1.67 (1.13–2.48)	.011	.535
NLRP3	rs10925027	N of the T allele	1.24 (0.96–1.60)	.101	.774	CT+TT vs CC	1.11 (0.74–1.65)	.621	.867	TT vs CC+CT	1.52 (1.06–2.19)	.023	.610
CARD8	rs2043211	N of the T allele	0.97 (0.75–1.24)	.783	.140	AT+TT vs AA	0.91 (0.64–1.29)	.593	.042	TT vs AA+AT	1.04 (0.66–1.66)	.860	.522

The results for each SNP were obtained by running separate regressions under the three genetic models indicated. Only malignant disease patients without previous transplantation history were included (N = 677). Excluded: CGVHD-unevaluable (N = 142) and day of CGVHD unknown (N = 3). The number of the primary competing events (ECGVHD) = 132. *P* and *P.xt* were obtained by the Wald test. Bold letters indicate $P < 0.005$ or $P.xt < 0.005$. Yellow highlighting indicates $P < 0.05$.

*Not applicable (N.A.) due to the violation of HWE. See the legend of Table 2 for other notations.

Table S10. Multivariable regressions of ECGVHD in all-disease patients

All patients without previous transplantation history (Group 1+2 in Supplementary Table S1)

	rs10925027		rs4612666	
	**SHR (95% CI)**	***P***	**SHR (95% CI)**	***P***
HLA-C MM	0.85 (0.44–1.65)	.632	1.22 (0.71–2.08)	.474
Donor *NLRP3* SNP, T-additive	1.08 (0.81–1.44)	.613	1.12 (0.83–1.52)	.445
HLA-C MM × Dn *NLRP3* SNP*	2.06 (1.32–3.21)	**.001**	1.55 (1.03–2.33)	.037
Recipient BMI†	1.75 (1.25–2.44)	**.001**	1.79 (1.28–2.50)	**<.001**
	P.xt = 0.486 (df = 4)		*P.xt* = 0.905 (df = 4)	

All patients (Group 1+2+3 in Supplementary Table S1)

	rs10925027		rs4612666	
	**SHR (95% CI)**	***P***	**SHR (95% CI)**	***P***
HLA-C MM	0.91 (0.49–1.67)	.751	1.24 (0.76–2.03)	.384
Donor *NLRP3* SNP, T-additive	1.10 (0.84–1.45)	.474	1.10 (0.83–1.47)	.497
HLA-C MM × Dn *NLRP3* SNP*	1.91 (1.25–2.90)	**.003**	1.50 (1.02–2.22)	.040
Recipient BMI†	1.58 (1.15–2.17)	**.0047**	1.61 (1.17–2.21)	**.003**
	P.xt = 0.577 (df = 4)		*P.xt* = 0.961 (df = 4)	

SH regressions were conducted fixing HLA-C MM, a donor *NLRP3* SNP, their interaction, and recipient BMI. In the upper two models, all (including non-malignant)-disease first-time transplantation patients were included (N = 737); CGVHD-unevaluable (N = 147) and day of CGVHD unknown (N = 3) were excluded. The number of primary competing events (ECGVHD) = 137. In the lower two models, all patients were included (N = 812); CGVHD-unevaluable (N = 184) and day of CGVHD unknown (N = 3) were excluded. The number of primary competing events (ECGVHD) = 152. *P* and *P.xt* were obtained by the Wald test.

*'Dn *NLRP3* SNP' stands for donor *NLRP3* SNP, referring to rs10925027 (left model) or rs4612666 (right model), under the T-additive model (TT vs CT vs CC). †High vs low+unknown (see Supplementary Table S1 for details). See the legend of Table 2 for other notations.

Table S11. SH regressions of ECGVHD fixing the interaction between HLA-C MM and donor *NLRP3* rs10925027, adjusted or unadjusted by reported risk factors

	Model 1		Model 2	
	**SHR (95% CI)**	***P***	**SHR (95% CI)**	***P***
HLA-C MM	0.83 (0.41–1.64)	.585	0.87 (0.44–1.71)	.682
Donor *NLRP3* rs10925027, T-additive	1.00 (0.73–1.35)	.978	0.99 (0.74–1.34)	.970
HLA-C MM × Dn rs10925027 Ta*	2.12 (1.32–3.38)	**.002**	2.09 (1.33–3.29)	**.001**
Recipient BMI, high vs low+unknown	1.77 (1.25–2.50)	**.001**		
Myeloablative, yes vs no	1.37 (0.85–2.19)	.194		
Disease stage†	0.99 (0.68–1.43)	.938		
Recipient age, high vs low	0.95 (0.66–1.37)	.782		
Donor age, high vs low	0.97 (0.69–1.38)	.884		
Female donor–male recipient‡	1.66 (1.13–2.45)	.010		
	P.xt = 0.624 (df = 9)		*P.xt* = 0.609 (df = 3)	

Model 1 includes reported risk factors for CGVHD or ECGVHD, in addition to the interaction between HLA-C MM and donor *NLRP3* rs10925027. Model 2 shows the unadjusted regression in relation to HLA-C MM, donor *NLRP3* rs10925027, and their interaction. Only malignant disease patients without previous transplantation history were included (N = 677). Excluded: CGVHD-unevaluable (N = 142) and day of CGVHD unknown (N = 3). The number of the primary competing events (ECGVHD) = 132. *P* and *P.xt* were obtained by the Wald test.

*‘Dn rs10925027 Ta’ stands for donor *NLRP3* rs10925027 under the T-additive model (TT vs CT vs CC). †Advanced+unknown vs standard. ‡vs female donor–female recipient, male donor–male recipient, and male donor–female recipient, combined. See the legend of Table 2 for other notations.

Table S12. Univariable Cox regression of overall survival

Recipient SNPs

		Additive model				Dominant model				Recessive model			
**Gene**	**SNP**		**HR (95% CI)**	***P***	***P.xt***		**HR (95% CI)**	***P***	***P.xt***		**HR (95% CI)**	***P***	***P.xt***
NLRP1	rs11651270*	N of the C allele	N.A.	N.A.	N.A.	CT+CC vs TT	N.A.	N.A.	N.A.	CC vs TT+CT	N.A.	N.A.	N.A.
NLRP2	rs1043673	N of the A allele	0.99 (0.83–1.17)	.867	.866	AC+AA vs CC	1.01 (0.82–1.25)	.927	.813	AA vs CC+AC	0.87 (0.55–1.37)	.546	.952
NLRP3	rs4612666	N of the T allele	1.04 (0.90–1.21)	.609	.261	CT+TT vs CC	1.11 (0.89–1.39)	.345	.053	TT vs CC+CT	0.96 (0.73–1.28)	.796	.679
NLRP3	rs10925027	N of the T allele	1.16 (1.00–1.35)	.047	.928	CT+TT vs CC	1.20 (0.94–1.53)	.134	.250	TT vs CC+CT	1.24 (0.98–1.57)	.080	.304
CARD8	rs2043211	N of the T allele	1.16 (1.01–1.35)	.040	.383	AT+TT vs AA	1.25 (1.00–1.55)	.047	.096	TT vs AA+AT	1.20 (0.92–1.57)	.188	.656

Donor SNPs

		Additive model				Dominant model				Recessive model			
**Gene**	**SNP**		**HR (95% CI)**	***P***	***P.xt***		**HR (95% CI)**	***P***	***P.xt***		**HR (95% CI)**	***P***	***P.xt***
NLRP1	rs11651270	N of the C allele	1.02 (0.87–1.20)	.778	.795	CT+CC vs TT	1.01 (0.82–1.24)	.946	.947	CC vs TT+CT	1.10 (0.77–1.59)	.595	.646
NLRP2	rs1043673	N of the A allele	1.15 (0.96–1.37)	.121	.373	AC+AA vs CC	1.10 (0.89–1.35)	.392	.500	AA vs CC+AC	1.63 (1.08–2.47)	.020	.386
NLRP3	rs4612666	N of the T allele	0.95 (0.82–1.11)	.523	.282	CT+TT vs CC	0.94 (0.76–1.17)	.570	.327	TT vs CC+CT	0.94 (0.71–1.24)	.640	.451
NLRP3	rs10925027	N of the T allele	0.96 (0.83–1.11)	.579	.738	CT+TT vs CC	0.85 (0.67–1.08)	.196	.555	TT vs CC+CT	1.04 (0.83–1.32)	.723	.271
CARD8	rs2043211	N of the T allele	0.99 (0.85–1.16)	.937	.118	AT+TT vs AA	0.95 (0.77–1.17)	.620	.056	TT vs AA+AT	1.08 (0.81–1.45)	.591	.760

The results for each SNP were obtained by running separate regressions under the three genetic models indicated. Only malignant disease patients without previous transplantation history were included (N = 822). Excluded: None. The number of the primary events (death) = 354. *P* and *P.xt* were obtained by the Wald test. Bold letters indicate $P < 0.005$ or $P.xt < 0.005$. Yellow highlighting indicates $P < 0.05$. HR stands for hazard ratio. See the legend of Table 2 for other notations.

*Not applicable (N.A.) due to the violation of HWE.

Table S13. Directed multivariable Cox regression of OS fixing recipient *NLRP3* rs4612666

	**HR (95% CI)**	***P***
Donor CMV serostatus, positive+unknown vs negative	1.15 (0.86–1.54)	.348
Recipient *NLRP3* rs4612666, C-recessive (CC vs CT+TT)	0.52 (0.32–0.83)	.007
Donor CMV × recipient *NLRP3* rs4612666, C-recessive	2.25 (1.31–3.85)	**.003**
Disease stage, advanced+unknown vs standard	1.77 (1.42–2.21)	**<.001**
Recipient age, high vs low	1.70 (1.37–2.12)	**<.001**
Recipient performance status, high vs low	1.48 (1.19–1.83)	**<.001**
	P.xt = 0.009 (df = 6)	

Only malignant-disease patients without previous transplantation history (Group 1 in Supplementary Table S1) were analyzed (N = 822). Excluded: None. The number of events (death) = 354. *P* and *P.xt* were obtained by the Wald test. The interaction term between donor CMV serostatus and recipient rs4612666 was retained throughout BIC-based variable selection (without fixation), when the non-interaction terms were fixed. Note that in contrast to Tables 4 and 5, the positive and unknown categories for donor CMV serostatuses were merged in this initial analysis.

Table S14. Multivariable Cox regressions of OS in relation to the interaction between recipient *NLRP3* SNP and donor CMV serostatus in patients with all diseases

	All first-time transplantation patients		All patients	
	**HR (95% CI)**	***P***	**HR (95% CI)**	***P***
Donor CMV, positive vs negative	1.15 (0.86–1.54)	.347	1.02 (0.78–1.32)	.890
Recipient rs4612666, C-recessive*	0.48 (0.30–0.77)	**.002**	0.42 (0.27–0.66)	**<.001**
Donor CMV × Rp rs4612666 Cr†	2.41 (1.41–4.12)	**.001**	2.49 (1.50–4.11)	**<.001**
Recipient age, high vs low	1.99 (1.60–2.47)	**<.001**	1.80 (1.48–2.19)	**<.001**
Performance status, high vs low	1.71 (1.39–2.11)	**<.001**	1.78 (1.47–2.16)	**<.001**
	P.xt = 0.050 (df = 5)		*P.xt* = 0.033 (df = 5)	

	All first-time transplantation patients		All patients	
	**HR (95% CI)**	***P***	**HR (95% CI)**	***P***
Donor CMV, positive vs negative	0.93 (0.62–1.40)	.730	0.87 (0.60–1.25)	.451
Recipient rs10925027, C-additive*	0.60 (0.44–0.81)	**<.001**	0.62 (0.47–0.81)	**<.001**
Donor CMV × Rp rs10925027 Ca‡	1.65 (1.17–2.33)	**.004**	1.57 (1.15–2.15)	**.004**
Recipient age, high vs low	2.02 (1.63–2.51)	**<.001**	1.83 (1.50–2.22)	**<.001**
Performance status, high vs low	1.69 (1.37–2.08)	**<.001**	1.76 (1.45–2.13)	**<.001**
	P.xt = 0.592 (df = 5)		*P.xt* = 0.643 (df = 5)	

In the left panels of both the upper and lower models, all-disease first-time transplantation patients (Group 1+2 in Supplementary Table S1) were included (N = 872). Excluded: Donor CMV serostatus unknown (*N* = 15). The number of events (death) = 358. In the right panels of both the upper and lower models, all patients (Group 1+2+3) were included (N = 980). Excluded: Donor CMV serostatus unknown (*N* = 19). The number of events (death) = 420. *P* and *P.xt* were obtained by the Wald test. The covariates adjusted are the same as used in the models shown in Tables 4 and 5, except that disease stage, which is undefined for non-malignant–disease patients, was not used as a covariate.

*These terms represent the effects of these recipient *NLRP3* SNPs in patients transplanted from CMV-negative donors, because CMV-negative status was coded as 0 in the model. †'Rp rs4612666 Cr' stands for the recipient *NLRP3* SNP, rs4612666, under the C-recessive model. ‡'Rp rs10925027 Ca'

stands for the recipient *NLRP3* SNP, rs10925027, under the C-additive model. See the legend of Table 2 for other notations.

Table S15. Association between donor and recipient CMV status

		Donor CMV status	
		**Negative**	**Positive**
**Recipient CMV status**	**Negative**	74	64
	**Positive**	160	456

Odds ratio (95% CI) = 3.30 (2.25–4.82). *P* < 0.001.

Table S16. Cox regressions of OS fixing the interaction between recipient *NLRP3* rs4612666 and donor CMV serostatus, adjusted or unadjusted by reported risk factors

	Model 1		Model 2	
	**HR (95% CI)**	***P***	**HR (95% CI)**	***P***
Donor CMV, positive vs negative	1.14 (0.85–1.53)	.390	1.21 (0.90–1.63)	.199
Recipient rs4612666, C-recessive	0.51 (0.32–0.83)	.006	0.54 (0.34–0.87)	.012
Donor CMV × Rp rs4612666 Cr*	2.20 (1.28–3.77)	**.004**	2.01 (1.18–3.45)	.011
Disease stage†	1.77 (1.42–2.22)	**<.001**		
Recipient age, high vs low	1.70 (1.36–2.13)	**<.001**		
Performance status, high vs low	1.49 (1.19–1.85)	**<.001**		
Donor age, high vs low	1.00 (0.81–1.24)	.987		
ABO blood type, mismatch vs match	1.05 (0.85–1.31)	.633		
HLA-C MM	1.15 (0.92–1.44)	.233		
Female donor–male recipient§	1.08 (0.82–1.41)	.601		
	P.xt = 0.011 (df = 10)		*P.xt* = 0.021 (df = 3)	

Model 1 includes reported risk factors for OS, in addition to the interaction between donor CMV serostatus and recipient *NLRP3* rs4612666. Model 2 shows the unadjusted regression in relation to the interaction between donor CMV serostatus and recipient *NLRP3* rs4612666. All malignant-disease patients without previous transplantation history (Group 1 in Supplementary Table S1) were included (N = 807). Excluded: Donor CMV serostatus unknown (N = 15). The number of events (death) = 346. *P* and *P.xt* were obtained by the Wald test.

*'Rp rs4612666 Cr' stands for recipient *NLRP3* rs4612666 under the C-recessive model (CC vs CT+TT). †Advanced+unknown vs standard. ‡Positive+unknown vs negative. §vs female donor–female recipient, male donor–male recipient, and male donor–female recipient, combined. See the legend of Table 2 for other notations.

Table S17. Cox regressions of OS fixing the interaction between recipient *NLRP3* rs10925027 and donor CMV serostatus, adjusted or unadjusted by reported risk factors

	**Model 1**		**Model 2**	
	**HR (95% CI)**	***P***	**HR (95% CI)**	***P***
Donor CMV, positive vs negative	0.88 (0.59–1.33)	.557	0.98 (0.65–1.48)	.937
Recipient rs10925027, C-additive*	0.60 (0.44–0.81)	**.001**	0.63 (0.47–0.86)	**.003**
Donor CMV × Rp rs10925027 Ca†	1.68 (1.18–2.39)	**.004**	1.56 (1.10–2.22)	.013
Disease stage‡	1.78 (1.42–2.23)	**<.001**		
Recipient age, high vs low	1.73 (1.38–2.17)	**<.001**		
Performance status, high vs low	1.46 (1.17–1.82)	**.001**		
Donor age, high vs low	1.01 (0.81–1.24)	.957		
ABO blood type, mismatch vs match	1.06 (0.85–1.32)	.586		
HLA-C MM	1.14 (0.91–1.43)	.247		
Female donor–male recipient§	1.09 (0.83–1.43)	.533		
	P.xt = 0.113 (df = 12)		*P.xt* = 0.903 (df = 3)	

Model 1 includes reported risk factors for OS, in addition to the interaction between donor CMV serostatus and recipient *NLRP3* rs4612666. Model 2 shows the unadjusted regression in relation to the interaction between donor CMV serostatus and recipient *NLRP3* rs10925027. Malignant-disease patients without previous transplantation history (Group 1 in Supplementary Table S1) were included (N = 807). Excluded: Donor CMV serostatus unknown (N = 15). The number of events (death) = 346. *P* and *P.xt* were obtained by the Wald test.

*This term represents the effects of this SNP in patients transplanted from CMV-negative donors, because CMV-negative and CMV-positive statuses were coded as 0 and 1, respectively, in the model.

†'Rp rs10925027 Ca' stands for recipient *NLRP3* rs10925027 under the C-additive model (CC vs CT vs TT). ‡Advanced+unknown vs standard. §vs female donor–female recipient, male donor–male recipient, and male donor–female recipient, combined. See the legend of Table 2 for other notations.

Table S18. Univariable SH regression of grade 3–4 AGVHD

Recipient SNPs

		Additive model				Dominant model				Recessive model			
**Gene**	**SNP**		**SHR (95% CI)**	***P***	***P.xt***		**SHR (95% CI)**	***P***	***P.xt***		**SHR (95% CI)**	***P***	***P.xt***
NLRP1	rs11651270*	N of the C allele	N.A.	N.A.	N.A.	CT+CC vs TT	N.A.	N.A.	N.A.	CC vs TT+CT	N.A.	N.A.	N.A.
NLRP2	rs1043673	N of the A allele	1.16 (0.82–1.64)	.398	.562	AC+AA vs CC	1.16 (0.74–1.80)	.519	.480	AA vs CC+AC	1.40 (0.65–3.01)	.391	.848
NLRP3	rs4612666	N of the T allele	1.11 (0.81–1.52)	.508	.337	CT+TT vs CC	1.14 (0.71–1.82)	.589	.400	TT vs CC+CT	1.17 (0.67–2.04)	.586	.538
NLRP3	rs10925027	N of the T allele	1.01 (0.75–1.36)	.933	.006	CT+TT vs CC	1.10 (0.66–1.83)	.707	.108	TT vs CC+CT	0.93 (0.56–1.57)	.795	.007
CARD8	rs2043211	N of the T allele	0.95 (0.70–1.30)	.762	.726	AT+TT vs AA	0.95 (0.61–1.48)	.815	.752	TT vs AA+AT	0.92 (0.50–1.70)	.789	.789

Donor SNPs

		Additive model				Dominant model				Recessive model			
**Gene**	**SNP**		**SHR (95% CI)**	***P***	***P.xt***		**SHR (95% CI)**	***P***	***P.xt***		**SHR (95% CI)**	***P***	***P.xt***
NLRP1	rs11651270	N of the C allele	0.94 (0.67–1.33)	.737	.623	CT+CC vs TT	0.91 (0.59–1.41)	.662	.938	CC vs TT+CT	1.00 (0.47–2.15)	.999	.139
NLRP2	rs1043673	N of the A allele	1.39 (0.99–1.96)	.056	.880	AC+AA vs CC	1.48 (0.96–2.29)	.079	.811	AA vs CC+AC	1.63 (0.72–3.69)	.244	.415
NLRP3	rs4612666	N of the T allele	0.83 (0.61–1.12)	.218	.053	CT+TT vs CC	0.89 (0.56–1.39)	.597	.211	TT vs CC+CT	0.59 (0.30–1.18)	.137	.030
NLRP3	rs10925027	N of the T allele	0.98 (0.73–1.31)	.875	.506	CT+TT vs CC	1.08 (0.64–1.82)	.783	.658	TT vs CC+CT	0.88 (0.53–1.45)	.611	.501
CARD8	rs2043211	N of the T allele	0.83 (0.60–1.15)	.271	.656	AT+TT vs AA	0.78 (0.50–1.22)	.280	.161	TT vs AA+AT	0.79 (0.41–1.54)	.495	.110

The results for each SNP were obtained by running separate regressions under the three genetic models indicated. Only malignant disease patients without previous transplantation history were analyzed (N = 787). Excluded: AGVHD-unevaluable (N = 34) and the day of grade 2/3/4 AGVHD unknown (N = 1). The number of the primary competing events (grade 3–4 AGVHD) = 80. *P* and *P.xt* were obtained by the Wald test. Bold letters indicate $P < 0.005$ or $P.xt < 0.005$. Yellow highlighting indicates $P < 0.05$.

*Not applicable (N.A.) due to the violation of HWE. See the legend of Table 2 for other notations.

Table S19. Univariable SH regression of overall CGVHD

Recipient SNPs

Gene	SNP	Additive model				Dominant model				Recessive model			
			SHR (95% CI)	*P*	*P.xt*		SHR (95% CI)	*P*	*P.xt*		SHR (95% CI)	*P*	*P.xt*
NLRP1	rs11651270*	N of the C allele	N.A.	N.A.	N.A.	CT+CC vs TT	N.A.	N.A.	N.A.	CC vs TT+CT	N.A.	N.A.	N.A.
NLRP2	rs1043673	N of the A allele	1.07 (0.86–1.33)	.530	.788	AC+AA vs CC	1.01 (0.78–1.31)	.927	.857	AA vs CC+AC	1.46 (0.90–2.38)	.127	.778
NLRP3	rs4612666	N of the T allele	0.97 (0.81–1.17)	.781	.555	CT+TT vs CC	0.90 (0.69–1.17)	.426	.608	TT vs CC+CT	1.09 (0.78–1.51)	.621	.684
NLRP3	rs10925027	N of the T allele	0.96 (0.79–1.15)	.638	.366	CT+TT vs CC	0.83 (0.63–1.09)	.171	.359	TT vs CC+CT	1.10 (0.82–1.49)	.526	.605
CARD8	rs2043211	N of the T allele	0.97 (0.81–1.17)	.782	.022	AT+TT vs AA	0.96 (0.74–1.25)	.776	.071	TT vs AA+AT	0.97 (0.67–1.40)	.876	.065

Donor SNPs

Gene	SNP	Additive model				Dominant model				Recessive model			
			SHR (95% CI)	*P*	*P.xt*		SHR (95% CI)	*P*	*P.xt*		SHR (95% CI)	*P*	*P.xt*
NLRP1	rs11651270	N of the C allele	0.81 (0.66–1.00)	.046	.100	CT+CC vs TT	0.80 (0.62–1.04)	.098	.190	CC vs TT+CT	0.63 (0.35–1.12)	.117	.256
NLRP2	rs1043673	N of the A allele	0.91 (0.73–1.13)	.383	.814	AC+AA vs CC	0.91 (0.70–1.19)	.499	.821	AA vs CC+AC	0.75 (0.37–1.50)	.412	.915
NLRP3	rs4612666	N of the T allele	1.23 (1.04–1.47)	.019	.757	CT+TT vs CC	1.29 (0.99–1.69)	.063	.872	TT vs CC+CT	1.37 (1.01–1.87)	.044	.468
NLRP3	rs10925027	N of the T allele	1.00 (0.84–1.19)	.979	.502	CT+TT vs CC	1.04 (0.77–1.41)	.790	.476	TT vs CC+CT	0.96 (0.71–1.28)	.764	.695
CARD8	rs2043211	N of the T allele	0.93 (0.77–1.12)	.456	.941	AT+TT vs AA	0.89 (0.68–1.15)	.358	.278	TT vs AA+AT	0.96 (0.67–1.38)	.824	.167

The results for each SNP were obtained by running separate regressions under the three genetic models indicated. Only malignant disease patients without previous transplantation history were analyzed (N = 677). Excluded: CGVHD-unevaluable (N = 142) and day of CGVHD unknown (N = 3). The number of the primary competing events (limited + extensive CGVHD) = 235. *P* and *P.xt* were obtained by the Wald test. Bold letters indicate $P < 0.005$ or $P.xt < 0.005$. Yellow highlighting indicates $P < 0.05$.

*Not applicable (N.A.) due to the violation of HWE. See the legend of Table 2 for other notations.

Table S20. Univariable SH regression of neutrophil engraftment

Recipient SNPs

		**Additive model**				**Dominant model**				**Recessive model**			
**Gene**	**SNP**		**SHR (95% CI)**	***P***	***P.xt***		**SHR (95% CI)**	***P***	***P.xt***		**SHR (95% CI)**	***P***	***P.xt***
NLRP1	rs11651270*	N of the C allele	N.A.	N.A.	N.A.	CT+CC vs TT	N.A.	N.A.	N.A.	CC vs TT+CT	N.A.	N.A.	N.A.
NLRP2	rs1043673	N of the A allele	1.05 (0.95-1.16)	.334	.387	AC+AA vs CC	1.06 (0.93-1.21)	.358	.689	AA vs CC+AC	1.06 (0.85-1.32)	.594	.149
NLRP3	rs4612666	N of the T allele	0.98 (0.90-1.08)	.735	.284	CT+TT vs CC	0.97 (0.85-1.11)	.623	.489	TT vs CC+CT	1.00 (0.85-1.18)	.996	.282
NLRP3	rs10925027	N of the T allele	1.04 (0.96-1.14)	.335	.282	CT+TT vs CC	1.10 (0.95-1.27)	.191	.852	TT vs CC+CT	1.01 (0.88-1.17)	.846	.041
CARD8	rs2043211	N of the T allele	0.94 (0.86-1.04)	.223	.338	AT+TT vs AA	0.97 (0.85-1.11)	.652	.223	TT vs AA+AT	0.85 (0.72-1.02)	.077	.753

Donor SNPs

		**Additive model**				**Dominant model**				**Recessive model**			
**Gene**	**SNP**		**SHR (95% CI)**	***P***	***P.xt***		**SHR (95% CI)**	***P***	***P.xt***		**SHR (95% CI)**	***P***	***P.xt***
NLRP1	rs11651270	N of the C allele	0.98 (0.88-1.08)	.663	.179	CT+CC vs TT	0.97 (0.85-1.10)	.600	.087	CC vs TT+CT	0.99 (0.78-1.26)	.936	.955
NLRP2	rs1043673	N of the A allele	0.95 (0.85-1.06)	.328	.565	AC+AA vs CC	0.98 (0.86-1.11)	.746	.497	AA vs CC+AC	0.75 (0.55-1.02)	.063	.769
NLRP3	rs4612666	N of the T allele	1.00 (0.91-1.09)	.930	.894	CT+TT vs CC	1.04 (0.91-1.19)	.607	.751	TT vs CC+CT	0.93 (0.78-1.11)	.427	.547
NLRP3	rs10925027	N of the T allele	1.01 (0.92-1.11)	.840	.680	CT+TT vs CC	1.05 (0.90-1.23)	.520	.585	TT vs CC+CT	0.98 (0.85-1.13)	.756	.907
CARD8	rs2043211	N of the T allele	1.01 (0.93-1.10)	.825	.759	AT+TT vs AA	0.99 (0.87-1.13)	.902	.390	TT vs AA+AT	1.05 (0.90-1.24)	.529	.047

Only malignant disease patients without previous transplantation history were analyzed (N = 822). Excluded: none. The number of the primary competing events (engraftment) = 782. Note that a risk factor for failed/delayed engraftment should exhibit SHR < 1. *P* and *P.xt* were obtained by the Wald test. Bold letters indicate *P* < 0.005 or *P.xt* < 0.005. Yellow highlighting indicates *P* < 0.05.

*Not applicable (N.A.) due to the violation of HWE. See the legend of Table 2 for other notations.

Table S21. Univariable SH regression of non-relapse mortality (NRM)

Recipient SNPs

Gene	SNP	Additive model				Dominant model				Recessive model			
			SHR (95% CI)	*P*	*P.xt*		SHR (95% CI)	*P*	*P.xt*		SHR (95% CI)	*P*	*P.xt*
NLRP1	rs11651270*	N of the C allele	N.A.	N.A.	N.A.	CT+CC vs TT	N.A.	N.A.	N.A.	CC vs TT+CT	N.A.	N.A.	N.A.
NLRP2	rs1043673	N of the A allele	0.86 (0.67–1.10)	.222	.613	AC+AA vs CC	0.81 (0.60–1.09)	.168	.674	AA vs CC+AC	0.89 (0.49–1.64)	.715	.647
NLRP3	rs4612666	N of the T allele	1.06 (0.86–1.31)	.572	.251	CT+TT vs CC	1.07 (0.78–1.45)	.683	.161	TT vs CC+CT	1.11 (0.76–1.61)	.592	.889
NLRP3	rs10925027	N of the T allele	1.22 (1.00–1.50)	.054	.832	CT+TT vs CC	1.34 (0.94–1.90)	.102	.646	TT vs CC+CT	1.28 (0.92–1.77)	.140	.433
CARD8	rs2043211	N of the T allele	1.07 (0.87–1.31)	.521	.812	AT+TT vs AA	1.10 (0.81–1.49)	.533	.473	TT vs AA+AT	1.08 (0.74–1.59)	.687	.457

Donor SNPs

Gene	SNP	Additive model				Dominant model				Recessive model			
			SHR (95% CI)	*P*	*P.xt*		SHR (95% CI)	*P*	*P.xt*		SHR (95% CI)	*P*	*P.xt*
NLRP1	rs11651270	N of the C allele	0.93 (0.74–1.17)	.531	.775	CT+CC vs TT	0.90 (0.68–1.21)	.492	.740	CC vs TT+CT	0.94 (0.55–1.60)	.818	.951
NLRP2	rs1043673	N of the A allele	1.01 (0.78–1.32)	.911	.691	AC+AA vs CC	0.95 (0.71–1.28)	.751	.717	AA vs CC+AC	1.40 (0.75–2.61)	.290	.808
NLRP3	rs4612666	N of the T allele	0.92 (0.74–1.13)	.404	.336	CT+TT vs CC	0.90 (0.67–1.22)	.505	.304	TT vs CC+CT	0.87 (0.58–1.29)	.485	.616
NLRP3	rs10925027	N of the T allele	0.94 (0.76–1.16)	.564	.876	CT+TT vs CC	0.84 (0.60–1.17)	.302	.488	TT vs CC+CT	1.01 (0.73–1.39)	.972	.686
CARD8	rs2043211	N of the T allele	1.02 (0.83–1.26)	.833	.249	AT+TT vs AA	0.98 (0.73–1.32)	.918	.060	TT vs AA+AT	1.12 (0.76–1.65)	.575	.720

The results for each SNP were obtained by running separate regressions under the three genetic models indicated. Only malignant disease patients without previous transplantation history were analyzed (N = 766). Excluded: no complete remission achieved after BMT (N = 56). The number of the primary competing events (NRM) = 182. *P* and *P.xt* were obtained by the Wald test. Bold letters indicate *P* < 0.005 or *P.xt* < 0.005. Yellow highlighting indicates *P* < 0.05.

*Not applicable (N.A.) due to the violation of HWE. See the legend of Table 2 for other notations.

Table S22. Univariable SH regression of relapse

Recipient SNPs

		Additive model				Dominant model				Recessive model			
**Gene**	**SNP**		**SHR (95% CI)**	***P***	***P.xt***		**SHR (95% CI)**	***P***	***P.xt***		**SHR (95% CI)**	***P***	***P.xt***
NLRP1	rs11651270*	N of the C allele	N.A.	N.A.	N.A.	CT+CC vs TT	N.A.	N.A.	N.A.	CC vs TT+CT	N.A.	N.A.	N.A.
NLRP2	rs1043673	N of the A allele	1.05 (0.82–1.34)	.709	.328	AC+AA vs CC	1.12 (0.81–1.54)	.501	.258	AA vs CC+AC	0.87 (0.44–1.69)	.672	.992
NLRP3	rs4612666	N of the T allele	0.99 (0.81–1.23)	.950	.659	CT+TT vs CC	1.19 (0.84–1.67)	.322	.928	TT vs CC+CT	0.72 (0.45–1.15)	.171	.448
NLRP3	rs10925027	N of the T allele	1.09 (0.88–1.35)	.449	.553	CT+TT vs CC	1.29 (0.88–1.89)	.189	.557	TT vs CC+CT	0.97 (0.66–1.42)	.861	.686
CARD8	rs2043211	N of the T allele	1.17 (0.93–1.46)	.171	.061	AT+TT vs AA	1.20 (0.86–1.66)	.286	.285	TT vs AA+AT	1.29 (0.86–1.95)	.218	.056

Donor SNPs

		Additive model				Dominant model				Recessive model			
**Gene**	**SNP**		**SHR (95% CI)**	***P***	***P.xt***		**SHR (95% CI)**	***P***	***P.xt***		**SHR (95% CI)**	***P***	***P.xt***
NLRP1	rs11651270	N of the C allele	1.09 (0.86–1.40)	.468	.033	CT+CC vs TT	1.10 (0.80–1.51)	.551	.087	CC vs TT+CT	1.18 (0.69–2.02)	.538	.032
NLRP2	rs1043673	N of the A allele	1.21 (0.93–1.57)	.157	.380	AC+AA vs CC	1.24 (0.90–1.71)	.182	.575	AA vs CC+AC	1.32 (0.67–2.59)	.420	.164
NLRP3	rs4612666	N of the T allele	1.19 (0.95–1.49)	.123	.544	CT+TT vs CC	1.26 (0.89–1.79)	.184	.380	TT vs CC+CT	1.26 (0.86–1.86)	.241	.938
NLRP3	rs10925027	N of the T allele	1.09 (0.87–1.36)	.475	.989	CT+TT vs CC	1.13 (0.77–1.68)	.527	.839	TT vs CC+CT	1.10 (0.77–1.56)	.593	.829
CARD8	rs2043211	N of the T allele	0.96 (0.76–1.21)	.737	.915	AT+TT vs AA	0.94 (0.68–1.30)	.705	.763	TT vs AA+AT	0.97 (0.62–1.51)	.894	.785

The results for each SNP were obtained by running separate regressions under the three genetic models indicated. Only malignant disease patients without previous transplantation history were analyzed (N = 766). Excluded: no complete remission achieved after BMT (N = 56). The number of the primary competing events (relapse) = 152. *P* and *P.xt* were obtained by the Wald test. Bold letters indicate $P < 0.005$ or $P.xt < 0.005$. Yellow highlighting indicates $P < 0.05$.

*Not applicable (N.A.) due to the violation of HWE. See the legend of Table 2 for other notations.

Table S23. Known SNPs in linkage disequilibrium with the five SNPs analyzed

SNP	N of nearby SNPs in LD at r^2 > 0.49*	N of nearby SNPs in LD at r^2 > 0.64*	Names of nearby SNPs in LD at r^2 > 0.64*
rs11651270 (*NLRP1*)	22	9	rs56750129, rs59564976, rs11870188, rs12937224, rs58604457, rs57636751, rs3786056, rs8074853, rs9896759
rs1043673 (*NLRP2*)	94	67	rs1654495, rs34990862, rs1654497, rs3837968, rs1671225, rs1671227, rs1654498, rs533591999, rs1671228, rs34679212, rs1654499, rs34890978, rs35700016, rs1654501, rs1654502, rs1654503, rs11881512, rs11881518, rs1671133, rs3786861, rs3786862, rs3786864, rs57854071, rs61098735, rs28403201, rs28542384, rs11667105, rs56720508, rs11667208, rs12982963, rs12982755, rs12982762, rs1043678, rs1043680, rs1043684, rs8110238, rs59012979, rs10402586, rs10401863, rs77062449, rs75062592, rs60906243, rs11666617, rs56874943, rs11670419, rs80231030, rs75513678, rs11881945, rs11881948, rs201955358, rs143674250, rs371050626, rs118028587, rs34167779, rs34539795, rs146092048, rs138843420, rs141529154, rs56988184, rs61527566, rs372118634, rs373859068, rs367825753, rs60969323, rs58989341, rs34584455, rs2304166
rs4612666 (*NLRP3*)	3	2	rs3806265, rs3835304
rs10925027 (*NLRP3*)	28	15	rs12143966, rs4925659, rs111307268, rs60823583, rs10159239, rs56383829, rs60831025, rs10754558, rs34691535, rs10802502, rs4925547, rs10754559, rs10733111, rs10733112, rs4925663
rs2043211 (*CARD8*)	54	46	rs3745717, rs2015209, rs2015211, rs2015216, rs36069776, rs11669584, rs11665831, rs11083924, rs7249075, rs7249085, rs7249785, rs71181660, rs1062808, rs4597433, rs71665156, rs4347733, rs4423519, rs71357807, rs11665981, rs1971784, rs1971783, rs71334276, rs1062806, rs1347170, rs2008527, rs3786739, rs12610708, rs7508437, rs3786742, rs34393028, rs10410274, rs10409908, rs10410871, rs16981829, rs12609126, rs11670750, rs11666976, rs11083925, rs2304134, rs10418189, rs9304675, rs8113696, rs71357810, rs6509365, rs6509366, rs16981853

*Only the same gene plus 100-kilobase up- and downstream sequences were searched in the data from 104 Japanese individuals in phase 3 of 1000 Genomes Project.

Figure S1. Representative genotyping results

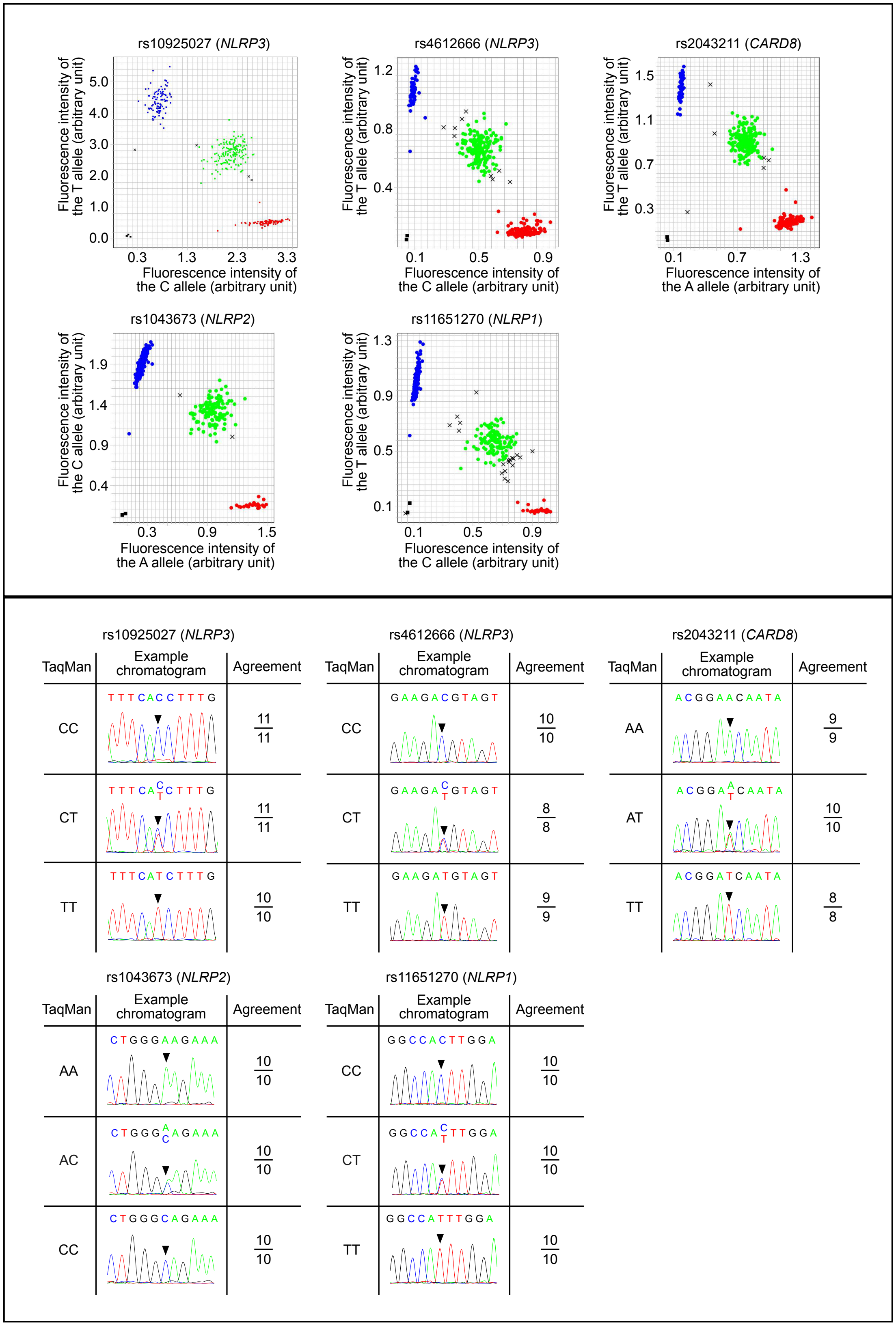

The upper panel shows allelic discrimination plot from one representative run of the TaqMan assay for each SNP drawn by the analysis software (Applied Biosystems). The red, blue, and green dots indicate the two homozygous genotypes and the heterozygous genotype, respectively. The X and black box represent the failed samples and the no-DNA control, respectively. The lower panel shows confirmation of TaqMan assays by PCR direct sequencing. Eight to 11 samples of each of the three genotypes successfully determined by the TaqMan assays were subsequently re-genotyped by PCR direct sequencing. The arrowhead indicates the position of each SNP.

Figure S2. Venn diagram of AGVHD-unevaluable status, CGVHD-unevaluable status, engraftment failure, and no achievement of complete remission after BMT

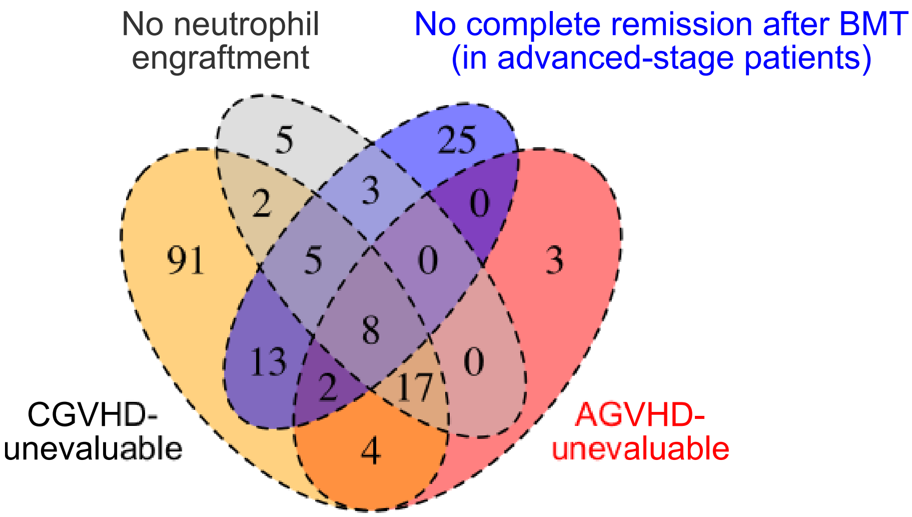

The 822 malignant-disease first-time transplantation patients were analyzed.

Supplementary Methods

Details of SNP selection

We chose two *NLRP3* SNPs (Supplementary Table S2). One is an intronic SNP, rs4612666, which is associated with susceptibility to food-induced anaphylaxis and aspirin-induced asthma in Japanese individuals[2]. The C allele creates a putative GATA-2 binding sequence, induces 1.2-fold higher expression of a downstream reporter gene, and affects an electrophoretic mobility shift assay pattern in THP-1 cells[2]. The other SNP is rs10925027, which is located downstream of both the *NLRP3* and *OR2B11* genes. This SNP was associated with relapse in an HLA-identical sibling HSCT study[4]. Although the molecular function of this SNP, if any, remains unknown, it is in strong LD in JPT104 from the 1000 Genomes Project[5] with a known functional SNP, rs10754558, which affects the stability of *NLRP3* mRNA[2]. The *NLRP1* SNP rs11651270 causes a missense substitution that is functionally important for autoproteolysis and IL-1β secretion, and is also associated with autoimmune diseases[1,6]. The *CARD8* SNP rs2043211 causes a nonsense substitution that abrogates inhibition of NF-κB activity by *CARD8* and is associated with disease severity in rheumatoid arthritis patients[3]. The *NLRP2* SNP rs1043673 causes a missense substitution of unknown molecular functional consequence and is associated with the harmful effects of arsenic[7]. This SNP was associated with NRM in a univariable analysis in the sibling HSCT study, and is in 100% LD in JPT104 with rs1043684, which was also associated with NRM and OS in the same study[4].

Details of SNP genotyping by direct sequencing

Short genomic regions spanning the two SNPs were amplified from 10 ng each of the DNAs by PCR using ExTaq HS (Takara), with the pairs of primers listed in Supplementary Table S2. The PCR products were treated with ExoSAP-IT (USB). DNA sequencing reactions were carried out using one of these primers and the BigDye Terminator v3.1 Cycle Sequencing kit, which was then read on the 3730xl DNA Analyzer (Applied Biosystems) at Eurofins Genomics (Tokyo). After visual inspection of each chromatogram, the ratio of peak amplitudes of the primary and secondary bases was calculated at each SNP nucleotide. The genotype was judged to be heterozygous for the primary and secondary bases if the ratio of the two peaks was less than 3, and otherwise judged as homozygous for the primary base.

Supplementary References

1 Finger, J. N. *et al.* Autolytic proteolysis within the function to find domain (FIIND) is required for NLRP1 inflammasome activity. *J. Biol. Chem.* **287**, 25030–25037 (2012).

2 Hitomi, Y. *et al.* Associations of functional NLRP3 polymorphisms with susceptibility to food-induced anaphylaxis and aspirin-induced asthma. *J. Allergy Clin. Immunol.* **124**, 779–785 (2009).

3 Fontalba, A. *et al.* Deficiency of the NF-kappaB inhibitor caspase activating and recruitment domain 8 in patients with rheumatoid arthritis is associated with disease severity. *J. Immunol.* **179**, 4867–4873 (2007).

4 Granell, M. *et al.* Common variants in NLRP2 and NLRP3 genes are strong prognostic factors for the outcome of HLA-identical sibling allogeneic stem cell transplantation. *Blood* **112**, 4337–4342 (2008).

5 The 1000 Genomes Project Consortium *et al.* A global reference for human genetic variation. *Nature* **526**, 68–74 (2015).

6 Levandowski, C. B. *et al.* NLRP1 haplotypes associated with vitiligo and autoimmunity increase interleukin-1beta processing via the NLRP1 inflammasome. *Proc. Natl. Acad. Sci. U. S. A.* **110**, 2952–2956 (2013).

7 Bhattacharjee, P. *et al.* Association of NALP2 polymorphism with arsenic induced skin lesions and other health effects. *Mutat. Res.* **755**, 1–5 (2013).
